# Striatal prediction errors support dynamic control of declarative memory decisions

**DOI:** 10.1038/ncomms13061

**Published:** 2016-10-07

**Authors:** Jason M. Scimeca, Perri L. Katzman, David Badre

**Affiliations:** 1 Department of Cognitive, Linguistic, and Psychological Sciences, Brown University, Providence, Rhode Island 02912, USA; 2 Helen Wills Neuroscience Institute, University of California, Berkeley, Berkeley, California 94720, USA; 3 Brown Institute for Brain Sciences, Brown University, Providence, Rhode Island 02912, USA

## Abstract

Adaptive memory requires context-dependent control over how information is retrieved, evaluated and used to guide action, yet the signals that drive adjustments to memory decisions remain unknown. Here we show that prediction errors (PEs) coded by the striatum support control over memory decisions. Human participants completed a recognition memory test that incorporated biased feedback to influence participants' recognition criterion. Using model-based fMRI, we find that PEs—the deviation between the outcome and expected value of a memory decision—correlate with striatal activity and predict individuals' final criterion. Importantly, the striatal PEs are scaled relative to memory strength rather than the expected trial outcome. Follow-up experiments show that the learned recognition criterion transfers to free recall, and targeting biased feedback to experimentally manipulate the magnitude of PEs influences criterion consistent with PEs scaled relative to memory strength. This provides convergent evidence that declarative memory decisions can be regulated via striatally mediated reinforcement learning signals.

## Abstract (web summary)

The human brain can efficiently retrieve information from long-term memory and use it to guide action but how the brain selects the most useful information in each case is unclear. Here the authors show that reinforcement learning mechanisms, based on expected value and prediction error fMRI signals in striatum, play a role in memory control processes guiding behavior.

## Graphical abstract

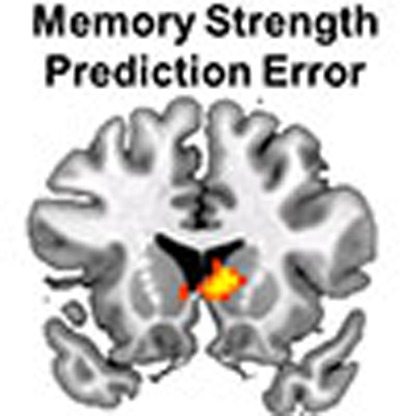

The human brain is capable of efficiently retrieving useful information from long-term memory and using this information to guide action. A challenge to any memory system is to retrieve and act on information that has high utility, given the current circumstances. The human memory systems have some intrinsic features related to basic encoding, retrieval, and consolidation that partly confront this information retrieval problem^1^,^2^. However, strategic cognitive control processes supported by frontoparietal brain networks can further shape retrieval by using current goals and context to adaptively guide retrieval toward high utility information and outcomes^3^,^4^,^5^,^6^,^7^. Memory disorders arising in patients with compromised cognitive control systems are often traceable to a failure of strategic guidance, evaluation and use of memory^8^. Yet a striking observation from both real life and the laboratory is that healthy individuals routinely apply memory retrieval strategies to positive effect, despite having little explicit training or instruction^1^,^3^,^9^,^10^,^11^. The declarative memory literature has identified a variety of control processes that guide retrieval, and the neural systems that implement this control are relatively well recognized^1^,^3^,^9^,^10^,^11^. However, a fundamental open question concerns how the brain acquires, evaluates, and adapts memory control processes. Here we show that reinforcement learning (RL) mechanisms can support the evaluation and adjustment of memory control processes.

Outside of the declarative memory domain, RL mechanisms are thought to contribute to the acquisition and adjustment of policies for strategic action selection. Frontostriatal circuits are central to this process, with frontal cortex supporting maintenance of task relevant information and the nigra–striatal dopamine system mapping value to the selection of appropriate actions and policies. Ventral striatum has been widely associated with receipt of primary and secondary rewards^12^,^13^ as well as reward prediction error (PE), which reflects the degree to which an experienced outcome value deviates from the expected value (EV). Across many experiments, these striatal value signals have been shown to drive learning of cognitive control for action selection and nondeclarative learning^12^,^14^,^15^,^16^,^17^.

Thus, one hypothesis is that cognitive control of declarative memory is similarly acquired through an RL (that is, nondeclarative learning) process, wherein positive PEs reinforce a reliance on a prevailing memory strategy and negative PEs punish the strategy^6^,^18^. Despite a large literature on RL and value-based decision-making, these nondeclarative learning processes are not typically considered in the context of declarative memory retrieval. Thus, connecting these divergent research areas may provide insight into the mechanisms that regulate cognitive control of memory retrieval. Further, declarative memory provides a novel domain in which to test the generalizability of RL principles. Importantly, however, the application of RL mechanisms to regulating cognitive control of declarative memory is complicated by several unknowns regarding how value is assigned to memory decisions and outcomes^18^,^19^,^20^,^21^.

We focus here on how human participants learn to relate evidence from memory to a decision and response; specifically, we address adjustments of the recognition memory criterion. Recognition memory involves classifying a stimulus as novel (new) or previously encountered (old). Recognition memory theory commonly assumes that all items will elicit at least some match to long-term memory and thus will provide some evidence of oldness, termed memory strength (MS)^5^,^22^,^23^,^24^,^25^,^26^. Old items (that is, items studied during learning) will have higher MS than new items (that is, unstudied items), although the distributions of MS for old and new items can overlap (Fig. 1a). The recognition decision criterion specifies the threshold level of evidence above which an old decision is made (Fig. 1a). Criterion setting is a form of mnemonic control in that it relates retrieved evidence to a context-dependent decision and response.

Having a dynamic recognition criterion provides the primary means by which an individual can balance errors due to misidentifying a new item as old (false alarms) or failing to recognize an old item (misses). A neutral criterion falls at the intersection of the studied and unstudied distributions (Fig. 1a). A conservative criterion requires more evidence for an old decision, while a liberal criterion requires less evidence for an old decision. The optimal criterion placement depends on an individual's context and goals^27^. For example, depending on whether you are at a professional conference or vacationing in a foreign country, you may be more or less likely to approach someone who is only vaguely familiar. The expectation that the person is someone you have met, the EV of speaking to them, and the costs of being wrong can all differ across these two scenarios. Thus, expectation and value arise at multiple stages in the retrieval and decision process.

One manipulation that produces dynamic adjustments in recognition criterion is biased feedback^28^. For example, providing false positive feedback following incorrect old responses biases the overall pattern of feedback and drives individuals to adopt a more liberal criterion. We used this paradigm to induce adjustments to decision criterion and identify the underlying neural mechanisms. We hypothesized that feedback elicits PE signals in striatum, and these PEs drive adjustments in recognition criterion by reinforcing or punishing the preceding memory decision. However, a challenge to testing this hypothesis is that the source of value associated with a memory decision is unknown. Using the framework of value-based decision-making models, we thus considered two alternative models of how the EV and PE are determined for memory decisions.

The first alternative, which we term the expected response outcome (ERO) alternative, holds that the EV of a memory decision is directly related to the expected outcome of the full trial following a particular response. As such, this EV can be estimated directly from the confidence in the memory decision. That is, a high confidence rating reflects a high expectation of getting the trial correct, and so receiving positive feedback. This relationship holds equivalently for both new and old responses. In signal-detection terms, higher confidence ratings correspond to memory decisions for items further from the criterion (Fig. 1a)^22^. Figure 1b shows four possible responses under this alternative. For example, positive feedback following a high confidence new response would result in a relatively small-positive PE and reinforce a modest conservative shift in criterion (Fig. 1b, top row).

The second alternative, which we term the MS alternative, holds that the EV of a memory decision is linked to the retrieved MS that led to that decision, such that greater retrieved MS is linked to greater EV. That is, more evidence of oldness confers higher EV regardless of the response that follows. Under this alternative, the EV of a decision is related directly to MS: while EV will be positively correlated with confidence ratings for old decisions, it will be negatively correlated with confidence for new decisions (Fig. 1a). Figure 1c shows four possible responses under this alternative. For example, positive feedback following an item with low MS (that is, a high confidence new response) would result in a relatively large positive PE and thus reinforce a large conservative shift in criterion (Fig. 1c, top row).

Both of these alternatives are couched in the framework of existing decision-making models and have plausible support from the existing memory literature. Individuals in recognition tasks typically demonstrate reliable metacognitive accuracy: high confidence responses are more likely to be followed by a positive outcome than are low confidence responses^22^,^29^. This is consistent with the ERO alternative and is largely analogous to the conceptualization of EV in conventional RL domains, in particular Q-learning models in which PE is response dependent^30^. The MS alternative is consistent with theories that argue that successful memory retrieval elicits approach or further processing (for example, familiarity gated retrieval)^31^,^32^ or that achieving the goal of retrieval is inherently rewarding^5^,^18^,^19^,^20^. Striatal activation is associated with retrieval success and has been linked to the rewarding character of memory retrieval^18^,^19^,^20^. Likewise, the source monitoring framework stresses the behavioural value of retrieving specific information from memory: memory is often probed in the service of action, and successful retrieval attempts give rise to positive outcomes (for example, searching memory for your mental grocery list while shopping)^5^. The MS alternative is similar to the conceptualization of value in actor-critic flavours RL, in which PE is computed relative to a state value rather than response-dependent value^30^.

We used the logic described in Fig. 1 to test the hypothesis that striatally mediated PEs drive adjustments in the recognition criterion. First, using fMRI and trial-by-trial estimates of MS-PEs and ERO-PEs, we test the hypothesis that striatum tracks PE and these PEs drive shifts in memory criterion. In a follow-up behavioural experiment, we target false feedback to different levels of confidence across experimental groups. This allows us to experimentally manipulate the magnitude of PEs elicited by false feedback and demonstrate that this manipulation influences the magnitude of criterion shifts across groups. Finally, using computational modelling and a free recall transfer protocol, we provide evidence that learning is localized to the evaluation of retrieved memory evidence, as opposed to a response bias.

## Results

### Biased feedback influences recognition memory criterion

Participants completed a recognition memory test during fMRI scanning (Fig. 2a; see Methods). For each test item, they made an old/new decision and indicated their confidence using a continuous confidence scale. Overall, participants accurately performed the task (mean proportion correct=76.41%; s.e.m. =1.21%) and fewer than 5% of trials were non-response errors. The mean response time was 993 ms (s.e.m.: 12 ms).

Following a jittered delay, positive (‘Correct!') or negative (‘Incorrect!') feedback was provided on each trial. False-positive feedback was provided on ∼70% of incorrect old responses (false alarms). All other responses received veridical feedback. This biased feedback drove participants to adopt a more liberal criterion over the course of the experiment (Fig. 2b). The criterion shift effect was supported by a main effect of time on criterion (F(3.3,59.9)=2.938, *P*=0.036, *η*_*P*_^2^=0.140), and the direction of the shift was confirmed by planned *t*-tests comparing the terminal criterion against the initial criterion (*t*(18)=2.848, *P*=0.011, Cohen's *d*=0.692) and against a neutral criterion of zero (*t*(18)=3.730, *P*=0.002, Cohen's *d*=0.859).

Whereas veridical feedback does not typically impact recognition criterion^28^,^33^, the biased feedback paradigm successfully drove changes in recognition decision criterion. Furthermore, the continuous confidence ratings and biased feedback protocol in the present experiment were designed to provide a wide range of PE values from trial to trial. Altogether, these features allow us to identify the neural correlates of PEs during memory decisions and link these neural PEs to behaviour.

### Striatum codes prediction errors for recognition decisions

**Figure 1:**
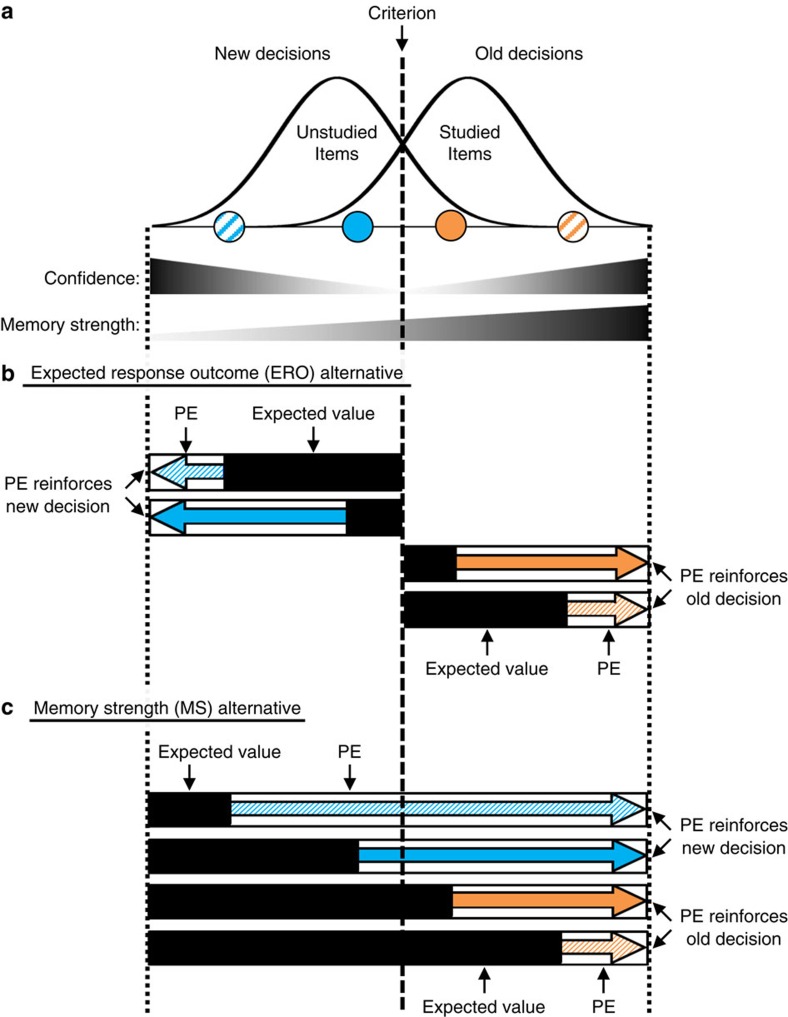
Two alternative models of computing the expected value (EV) and prediction errors (PE) for memory decisions. (**a**) Standard signal detection model of recognition memory decisions depicting memory strength distributions for unstudied (new) and studied (old) test items. Memory strength increases along the horizontal-axis from left to right; confidence increases for responses further from the criterion. The neutral criterion shown here falls at the intersection of the memory strength distributions for unstudied and studied items. The four circles correspond to four example memory test items of varying memory strength. The scale for confidence and memory strength depicted in **a** is directly mapped onto the scales for EV in **b** and **c**. (**b**) The EVs (black bars) for responses to the four example items under the expected response outcome alternative. EV values under this alternative are derived directly from the confidence ratings given for each test item and the magnitude of the black bars corresponds to the magnitude of the EV. (**c**) The EVs for responses to the same four items under the Memory Strength alternative. EVs under this alternative are derived from the memory strength associated with each test item and the magnitude of the black bars corresponds to the magnitude of the EV. (**b**,**c**) PEs are calculated as the difference between the feedback outcome and the EV. The PEs depicted here (coloured arrows) correspond to the magnitude of positive PEs following a positive feedback outcome on each example trial. Blue arrows correspond to positive PEs following a new decision and thus reinforce a more conservative criterion. Orange arrows correspond to positive PEs following an old decision and thus reinforce a more liberal criterion. The degree to which each positive (or negative) feedback instance will reinforce (or punish) a particular decision depends on the magnitude of the PE. On each trial, the EV and PE values were calculated using as input the old/new decision, confidence, and feedback outcome. See the Methods section for details regarding specific calculations for EV and PE values under the ERO and MS alternatives.

To identify PE signals in the brain we used the old/new response, confidence rating and feedback outcome for each trial to calculate trial-by-trial EVs and PEs under the logic of the MS and ERO alternatives (Fig. 1; see Methods). EVs and PEs were calculated for all trials on which participants made a decision, including false feedback and veridical feedback trials. We constructed a separate general linear model (GLM) for each of the ERO and MS alternatives using the respective EV and PE values generated from each alternative (Methods). This approach allows the regressors in each GLM to capture both the shared and unique variance for each alternative.

We first considered the neural circuitry that tracked trial-by-trial MS-PEs at feedback. As predicted, the striatum, along with other brain areas, tracked MS-PEs following memory decisions (Fig. 3b; Supplementary Fig. 1; Supplementary Table 1). The signal across an anatomical region-of-interest (ROI) encompassing the whole striatum (Methods) was significantly associated with MS-PE (*t*(18)=4.858, *P*<0.001, Cohen's *d*=1.114). Next, we tested whether striatal PEs are related to behaviour in the recognition test. The striatal signal from the ROI predicted individuals' terminal criteria (correlation between MS-PE parameter estimates and terminal criterion: *R*=−0.547, *P*=0.015; Fig. 4a), such that individuals with greater PE-related activation of striatum had more liberal criteria at the end of the experiment.

We found a similar pattern of results for the neural correlates of ERO-PEs. ERO-PE also activated striatum, among other brain regions (Supplementary Fig. 1; Supplementary Table 2). However, as will be discussed below, this similar pattern is likely due to the large degree of shared variance between the MS-PE and ERO-PE predictors (Fig. 3a).

### Memory strength PEs are linked to striatal signal

We constructed a third GLM that included both MS-PE and ERO-PE regressors (Methods). This approach allows the regressors to compete for variance and thus activity associated with either regressor in this model will be due to the unique variance above and beyond their shared variance. We found that the unique variance of MS-PE was tracked by striatum (Fig. 3c; Supplementary Fig. 1; Supplementary Table 1). The unique variance of ERO-PE was not significantly associated with any activity in striatum. Instead, the unique variance of ERO-PE was associated with activation in bilateral inferior parietal cortex and right inferior frontal gyrus (Fig. 3d; Supplementary Fig. 1; Supplementary Table 1).

We complemented this voxelwise analysis with ROI analyses. We first extracted the signal from the anatomical striatum ROI (Fig. 3e). Activity across the entire striatal ROI was significantly associated with unique MS-PE (*t*(18)=4.172, *P*<0.001, Cohen's *d*=0.957) but not with unique ERO-PE (*t*(18)=1.162, *P*=0.260, Cohen's *d*=0.266), although the difference between the two predictors did not reach significance (*t*(18)=1.558, *P*=0.137, Cohen's *d*=0.357). Then, to assess the pattern of unique variance across the striatum relative to other regions in the brain, we tested for a predictor × region interaction^34^. Guided by the functional activations observed for unique ERO-PE, we used the AAL database to construct a second anatomically defined ROI by combining bilateral inferior parietal masks with a mask of right inferior frontal gyrus opercularis (Fig. 3f). Activity in this anatomical ROI was not significantly associated with unique MS-PE (*t*(18)=−0.224, *P*=0.825, Cohen's *d*=−0.051); although there was a reliable association with unique ERO-PE (*t*(18)=3.355, *P*=0.004, Cohen's *d*=0.770) and a reliable difference between ERO-PE and MS-PE (*t*(18)=2.114, *P*=0.049, Cohen's *d*=0.827). We submitted the parameter estimates for unique MS-PE and unique ERO-PE from these two ROIs to a 2 × 2 ANOVA and found a significant predictor × ROI interaction (F(1,18)=13.224, *P*=0.002, *η*_*P*_^2^=0.424). Taken together, these results show that the signal in striatum is better explained by the MS-PE predictor than by the ERO-PE predictor, relative to the opposite pattern observed in frontoparietal regions.

This result is bolstered by several control analyses addressing the relative pattern of predicted PEs within old and new responses (see Methods; Supplementary Fig. 2). MS-PE is associated with striatal activity even after controlling for the MS alternative's prediction that PEs for positive feedback will always be larger following old than following new responses (compare orange versus blue arrows in Fig. 1c). Similarly, MS-PE is associated with striatal activity even when constraining the analysis to only feedback following new responses, when the MS and ERO alternatives make categorically opposite predictions (compare pattern of blue arrows in Fig. 1b,c).

### Expected value is coded by both retrieval and value regions

We found that the MS-EV regressor of shared and unique variance was coded in a broad network that included precuneus, medial prefrontal cortex (PFC), caudate, posterior cingulate and amygdala (Supplementary Fig. 3; Supplementary Table 2). This network overlapped with the ‘core' recollection network^20^,^35^,^36^ and regions typically exhibiting retrieval success effects^4^,^18^,^36^. Many of these regions have also been implicated in representing subjective value during choice tasks^37^,^38^,^39^. The ERO-EV regressor of shared and unique variance was correlated with a similar network of regions (Supplementary Fig. 3; Supplementary Table 2), overlapping with regions identified in previous studies of recognition confidence^29^. As a control analysis, we conducted the standard retrieval success contrast (correct old>correct new responses), which allows for comparison between the activation in this contrast to the activation associated with the various EV and PE signals (see Methods; Supplementary Fig. 2).

When we included MS-EV and ERO-EV in the same analysis (GLM 4; see Methods; Supplementary Fig. 3; Supplementary Table 2), we found that the unique variance of these regressors was represented in overlapping areas of ventromedial PFC (vmPFC) and precuneus^38^. ERO-EV, but not MS-EV, was represented in the left anterior frontal pole, left superior frontal gyrus, and left putamen. MS-EV, but not ERO-EV, was represented in a more posterior portion of vmPFC.

### Targeting feedback to manipulate the magnitude of PEs

**Figure 2:**
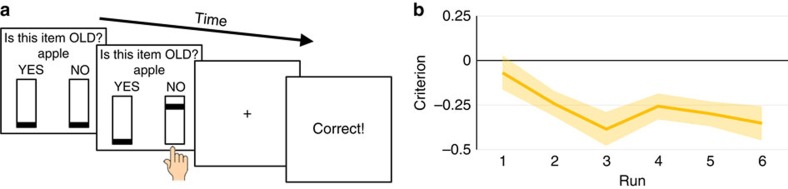
Behavioural task and results from fMRI experiment. (**a**) Participants completed a recognition memory test during fMRI scanning. On each trial, they made an old/new decision by pressing one of two buttons. They indicated their confidence using a continuous confidence scale: holding down the response button caused a black bar to move within the rectangle below their chosen response. They released the button to lock-in their confidence rating such that high confidence corresponded to the top of the rectangle. Positive (‘Correct!') or negative (‘Incorrect!') feedback was provided on each trial following a jittered fixation delay. In the fMRI experiment, false positive feedback was provided on ∼70% of incorrect old responses. Veridical positive and negative feedback was provided on all other trials. (**b**) The biased feedback paradigm elicited a liberal shift in criterion (that is, more negative criterion values) over the course of the recognition test. Error bars are s.e.m.

**Figure 3:**
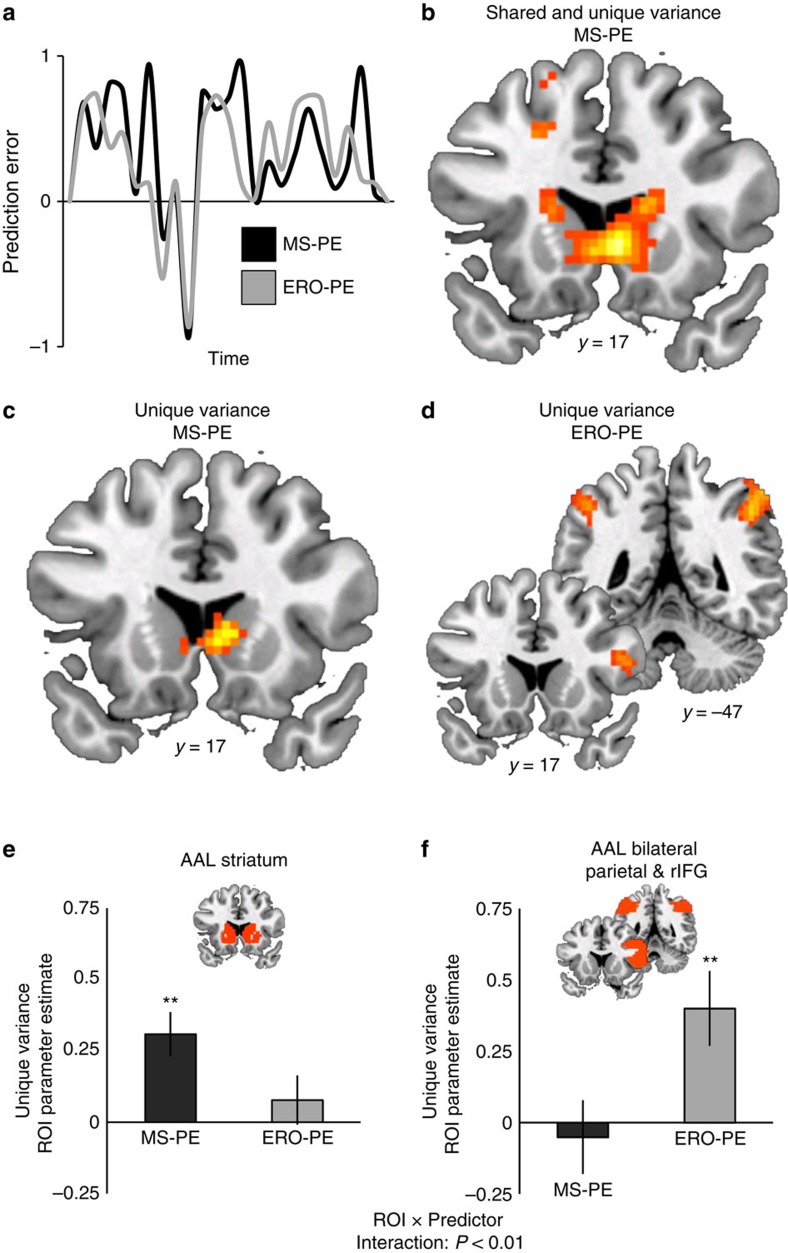
FMRI responses to trial-by-trial PE estimates. (**a**) Representative example of fMRI BOLD signal for PEs predicted by the memory strength (MS-PE) and expected response outcome (ERO-PE) alternatives. The cartoon signal displayed here represents the predicted BOLD signal over the course of 10 trials. The two alternatives make correlated but dissociable predictions. (**b**) Striatum tracks the trial-by-trial PE associated with the shared and unique variance of the memory strength alternative. (**c**,**d**) When the predictors are restricted to unique variance, MS-PE is coded in striatum and ERO-PE is coded in right inferior frontal gyrus and bilateral inferior parietal cortex. (**e**,**f**) Signal extracted from anatomical regions of interest (ROIs; shown inset; see text for definitions) reveal a significant ROI × predictor interaction. Whole-brain statistical maps are thresholded at *P*<0.05, FDR cluster corrected. Colour maps represent *t-*statistics with a range from 0 to 8. Parameter estimates are in arbitrary units. Error bars are s.e.m. **P<0.01 versus zero.

The link between MS-PEs, striatal activation, and individuals' terminal criterion provides support for the role of PEs in regulating recognition criterion and favours the MS alternative. In a follow-up between-groups behavioural experiment, we used the logic of Fig. 1 to test the hypothesis that targeting false positive feedback to specific levels of confidence will result in either small or large PEs, which will in turn drive small or large shifts in criterion. Participants in this experiment also completed a recognition memory test with a continuous confidence rating and biased feedback (see Methods; Fig. 2a). Although all instances of positive and negative feedback result in a PE, targeting false feedback allows us to experimentally bias the overall pattern of PE experienced by each group.

Importantly, the design allows us to differentiate between the different behavioural patterns predicted by the ERO and MS alternatives (Fig. 5a,b). Two groups received false positive feedback following incorrect new responses. Because this feedback reinforces the preceding decision, we predicted this would reinforce new decisions and result in a more conservative criterion^28^. Critically, one group received false feedback targeted to high-confidence new errors (for example, striped-blue circle in Fig. 1a; new—High Confidence group) and the other received false feedback targeted to low-confidence new errors (for example, solid-blue circle in Fig. 1a; New–Low Confidence group). The two alternatives make different predictions regarding the average magnitude of positive PEs elicited in each group. The ERO alternative predicts larger PEs and a more conservative criterion for the New–Low Confidence group (relative magnitude of blue arrows in Fig. 1b and Fig. 5a), as positive feedback following low confidence would elicit larger PEs. In contrast, the MS alternative predicts larger PEs and a more conservative criterion for the New–High Confidence group (relative magnitude of blue arrows in Fig. 1c and Fig. 5b), as positive feedback following low-MS would elicit larger PEs.

Two groups received false positive feedback following incorrect old responses, similar to the fMRI experiment, which we predicted would result in a liberal criterion^28^. Critically, one group received false feedback targeted to low-confidence old errors (for example, solid-orange circle in Fig. 1a; Old–Low Confidence group) and the other received false feedback targeted to high-confidence old errors (for example, striped-orange circle in Fig. 1a; Old–High Confidence group). Both alternatives predict that false feedback will elicit larger PEs for the Old–Low Confidence group than for the Old—High Confidence group (relative magnitude of orange arrows in Fig. 1b,c) and result in a more liberal criterion in the Old–Low Confidence group (Fig. 5a,b).

Figure 5 shows the terminal criterion for the four false-feedback groups and a control group that received completely veridical feedback on all trials. Our results are consistent with the MS alternative: the larger magnitude criterion are observed in the New—High Confidence and Old–Low Confidence groups. We submitted the terminal criterion values from the four biased feedback groups to a two-way analysis of variance (ANOVA) with factors of targeted response group (old/new) and targeted confidence (low/high). In support of the MS alternative, we found a main effect of targeted confidence (F(1,60)=6.612, *P*=0.013) and no interaction between the factors (F(1,60)=0.901, *P*=0.346). Note that the effect of PEs under the MS alternative manifests as a main effect because the two High Confidence groups show more positive criteria than the two Low Confidence groups (striped arrows/bars are more positive than solid arrows/bars in Fig. 5b,c). The two alternatives differ specifically in their predictions for the two New groups and a comparison between these groups confirmed that the New–High Confidence group adopted a more conservative criterion (*t*(30)=2.622, *P*=0.014), bolstering support for the MS alternative.

Control analyses confirmed that this result could not be explained by changes in response confidence between groups (Supplementary Note 1; Supplementary Table 3). Analysing criterion as a function of time revealed that initial learning occurs rapidly and continues throughout the test phase (Supplementary Note 1; Supplementary Fig. 4). Finally, an individual differences analysis that took into account the PE across all trials (veridical and false feedback) found that this net PE metric was correlated with participants' terminal criteria (Supplementary Note 1; Supplementary Fig. 5).

### The psychological locus of learning is in memory evaluation

Because the criterion measure in signal-detection theory is based on false alarms and misses, differences in criterion reflect differences in the proportions of old and new responses. As such, there are (at least) two possible psychological loci where learning could occur. Consider the liberal shift observed in the fMRI experiment: one explanation is that the adjustment simply involved an increased propensity to make old responses because old responses more frequently led to positive-feedback outcomes. Thus, individuals learned to bias their responding towards old responses regardless of the retrieved memory evidence, akin to the learning observed in typical RL and decision-making paradigms. We term this response-level learning. An alternative explanation is that the more liberal criterion reflects adjustments to the evaluation of retrieved memory evidence. That is, individuals learned to adjust the threshold above which they considered a given level of memory evidence as old. We term this memory-level learning. (This logic can likewise be applied to the more conservative criterion observed in the New groups of the behavioural experiment.) Thus, although the imaging and behavioural data suggest that memory-level PE is the learning signal used to regulate criterion, it remains important to test whether changes in criterion actually reflect the adjustment of memory process (that is, the interpretation of evidence from memory) versus motor or response learning.

To probe the psychological locus of learning, we applied computational modelling to the behavioural data collected during the fMRI experiment and separately to the data from the follow-up behavioural experiment. A criterion shift due to either memory-level or response-level learning will have the same result on response proportions (increased proportion of old or new responses) and thus standard signal-detection theory analyses cannot distinguish between these two alternatives. However, the drift diffusion model (DDM) framework accounts for both response proportions and the distribution of response times associated with response types^26^,^40^, and includes separate parameters that correspond to the memory-level learning and response-level learning alternatives^26^,^40^.

In the DDM, drift criterion (dc) determines whether a given level of retrieved evidence is considered evidence of oldness or evidence of newness. The response bias (*z*) parameter determines whether an old or a new response is more likely, regardless of the level of evidence. As such, shifts in dc or *z* map to the memory-level versus response-level accounts of criterion shift, respectively. These parameters are identifiable because they make distinct predictions regarding the response time distributions for old and new responses^26^ (see Methods for more details regarding these DDM parameters).

We first applied two DDM models to the data from the fMRI experiment: one in which the drift criterion was allowed to vary across participants and over time (memory-level learning), and one in which response bias was allowed to vary across participants and over time (response-level learning). The Drift Criterion model provided a better fit to the data (see Methods; Supplementary Table 4; Supplementary Fig. 6). Using the same logic, we applied a Drift Criterion model and Response Bias model to the data from the behavioural experiment. Again, the Drift Criterion model provided a better fit to the data (see Methods; Supplementary Table 4).

**Figure 4:**
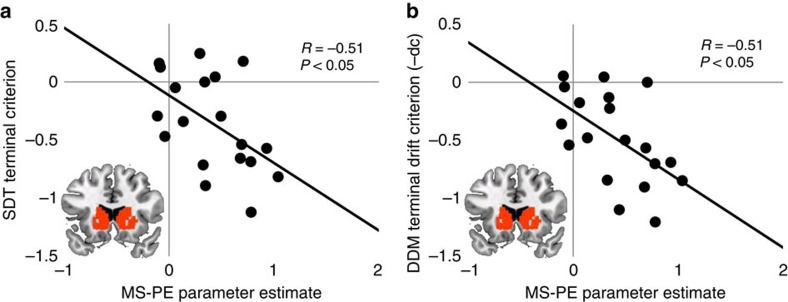
Correlation of striatal PE with terminal criterion and DDM drift criterion. Memory strength-Prediction Error (MS-PE) parameter estimates from striatum predict individuals' terminal criterion estimated from signal-detection theory (SDT; **a**) and terminal drift criterion estimated from drift diffusion modelling (DDM; **b**). In both panels, liberal criterion values correspond to negative values on the vertical axis. Parameter estimates are in arbitrary units and reflect the contribution of both shared and unique variance for MS-PE to BOLD signal in striatum.

**Figure 5:**
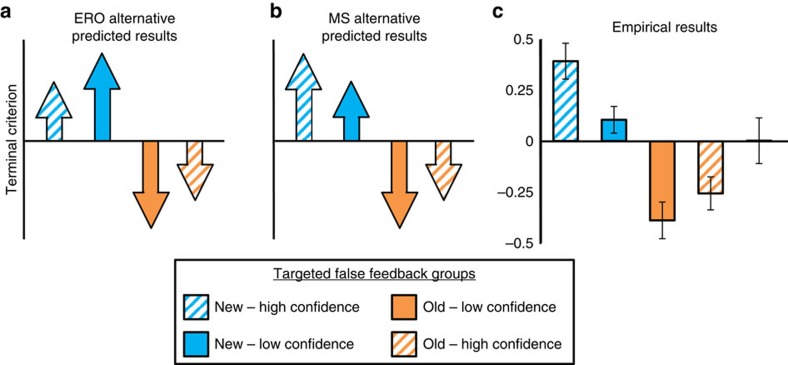
Targeting false feedback to manipulate the magnitude of PEs: logic and results. In a between-groups behavioural experiment, participants completed a recognition memory test in which they made an old/new decision and provided a continuous confidence rating. Veridical feedback was provided on the majority of trials. Probabilistic false positive feedback was targeted to specific response types (new or old errors) and levels of confidence (high or low confidence) across four groups. The expected response outcome (ERO) and memory strength (MS) alternatives make distinct predictions about the relative magnitude of PEs elicited by positive feedback targeted to different confidence levels for each response type (*cf.* Fig. 1). Thus, the two alternatives make distinct predictions about the relative magnitude of criterion shifts for the four groups, shown in **a** and **b**. Both alternatives predict that the two groups that received false feedback on new responses will show a conservative criterion (blue arrows) and that the two groups that received false feedback on old responses will show a liberal criterion (orange arrows). Any difference in criterion between the Low Confidence and High Confidence groups provides evidence consistent with a role for PEs in regulating recognition decisions. The specific pattern of results across the groups, specifically between the New–Low Confidence and New–High Confidence groups, differentiates between the two alternatives. (**c**) The empirical terminal criterion values from the four false-feedback groups. A control group that received completely veridical feedback adopted a neutral criterion, shown at the far right. Liberal criterion values correspond to negative values on the vertical axis. Error bars are s.e.m.

Finally, in the imaging experiment, the MS-PE striatal signal (shared and unique variance) across participants was correlated with the terminal drift criterion values estimated from the Drift Criterion model (*R*=0.55, *P*=0.016; Fig. 4b). Taken together, this replicates the DDM results across two independent data sets, indicating that shifts in drift criterion better fit the behavioural data and are closely linked to the MS-PE signal in the brain. We note that this does not rule out the possibility of a simultaneous and complementary role for response-level learning in recognition (see Methods; Supplementary Fig. 7). Furthermore, here we interpret the drift criterion parameter as reflecting an evaluation process that monitors retrieved evidence^26^,^41^ but the specific psychological interpretation of changes in drift criterion via different experimental manipulations remains an area of active research^26^,^41^,^42^,^43^,^44^.

### Transfer of recognition memory criterion to free recall

To further distinguish between response-level and memory-level learning and to demonstrate the generalizability of learning, we conducted a follow-up behavioural experiment to test whether learning that occurs during the recognition test extended to mnemonic evaluation processes involved in monitoring free recall performance. An individual's recognition criterion is predictive of that individual's false recall rate (that is, critical lure recall) in the Deese–Roediger–McDermott (DRM) false memory paradigm^45^. This suggests that evaluating memory evidence during recognition memory decisions and monitoring freely recalled items for output can tap a shared underlying evaluative process. For example, participants may generate candidate recall responses and then assess their associated MS, verbally reporting those responses that have sufficient evidence for oldness.

If the criterion results we observe in the biased-feedback recognition paradigm are due simply to changes in response bias (response-level learning), then terminal recognition criterion does not reflect memory evaluation, *per se*, and therefore would not predict false recall rates. However, because the neuroimaging and computational modelling suggest that regulating criterion involves adjustments in a memory evaluation process (memory-level learning), we predicted that terminal criterion following biased-feedback-induced shifts would successfully predict false recall rates.

Two groups of participants completed a recognition memory task. One group received biased feedback that induced a liberal criterion and one group received biased feedback that induced a conservative criterion (see Methods; Supplementary Fig. 8). Immediately following the recognition test, participants completed a free recall task for ten lists, each comprising words semantically related to a critical lure word that was omitted from the study list.

**Figure 6:**
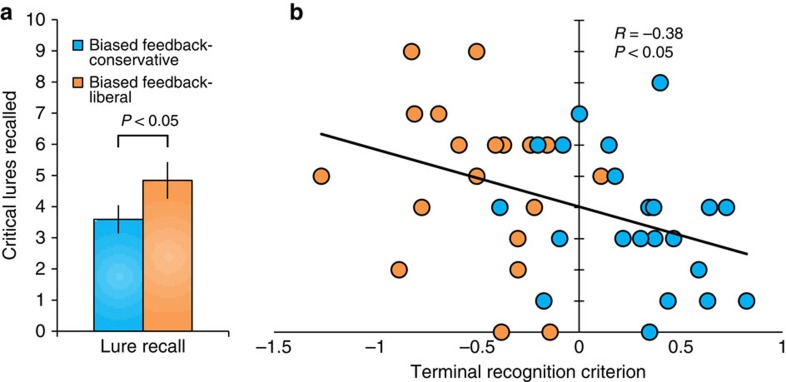
Performance in experiment testing transfer from recognition to free recall. (**a**) Mean critical lure recall for two groups of participants that completed a free recall false memory paradigm immediately after a recognition test with biased feedback that induced either a conservative (Biased Feedback–Conservative) or liberal (Biased Feedback–Liberal) criterion. The group that learned a conservative recognition criterion recalled fewer critical lures than the group that learned a liberal recognition criterion. *P* value shown for one-tailed *t*-test between groups. Error bars are s.e.m. (**b**) Terminal Recognition Criterion at the end of the biased-feedback recognition paradigm predicted the number of critical lures recalled.

We found that terminal criterion in the biased-feedback recognition task predicted critical lure recall: participants who adopted a more liberal criterion recalled more critical lures (*R*=−0.385, *P*=0.013; Fig. 6). In addition, we found that mean lure recall differed between groups as predicted: the group biased towards a liberal criterion was more likely to recall critical lures than the group biased towards a conservative criterion (*t*(39)=1.706, one-tailed *P*=0.048, Cohen's *d*=0.531; Fig. 6).

## Discussion

Here we provide convergent evidence that memory control strategies can be learned through RL mechanisms that rely on EV and PE signals deriving from memory retrieval itself. Specifically, we found that striatum tracked a trial-by-trial PE signal following feedback in a recognition memory test, with the PE signal scaled relative to the strength of retrieved memory evidence. This striatal PE signal was predictive of individuals' final criteria in the biased-feedback recognition test. Next, we targeted false feedback to different levels of confidence in order to experimentally manipulate the magnitude of PEs elicited by false feedback. This manipulation drove criterion in a pattern consistent with PEs scaled relative to MS. The behaviour from each of these experiments was best fit by a computational model in which learning occurred at the level of evidence evaluation rather than the level of response bias. Finally, we observed that the criterion learned during recognition transferred to free recall. The computational modelling and free recall results are both consistent with the idea that a general process used to evaluate mnemonic evidence is adjusted during recognition, rather than a specific response tendency.

These results advance an emerging literature on facilitative interactions between nondeclarative and declarative memory systems^6^,^18^,^46^,^47^,^48^. Although previous work has focused primarily on interactions during encoding^46^, the current study provides empirical evidence that the nondeclarative system also influences cognitive control of declarative memory retrieval. Specifically, value derived from memory outcomes could be one means by which mnemonic control strategies are learned. To our knowledge, the present study provides the first direct evidence that learning of control processes during memory retrieval can be mediated by PEs coded by the nigra–striatal system.

The results from the fMRI experiment and the targeted-feedback experiment provide converging evidence for the functional form by which memory retrieval confers value: the strength of retrieved memory was inherently valued, and PEs derived from this MS signal (MS-PEs) drove striatally mediated PE responses. By contrast, there was little evidence that the ERO-PE drove behaviour or striatal PE responding beyond the variance this signal shared with MS-PE. Although perhaps counterintuitive from the perspective of some RL models in which PE is response-dependent, the idea that MS would be inherently valued is consistent with prior research. For example, mere exposure effects have been widely observed in social psychology, wherein people show preference for stimuli they have consistently encountered previously^49^. In addition, general recognition of an object often triggers further retrieval^31^,^32^. Put another way, evidence of oldness elicits a type of mnemonic approach response. This value can provide a learning signal.

Consistent with the conclusion that value was mnemonic in origin, the MS-EV signal correlated with a set of regions that overlapped with regions in the core retrieval network^20^,^35^,^36^. Among these were activations in ventromedial frontal areas previously associated with meta-mnemonic feelings-of-knowing^50^ and memory schemas^51^. A speculative possibility is that initial retrieval of evidence from long-term memory results in a schema match/activation, and it is this congruency between retrieval outcome and a prevailing schema representation that is valued. This hypothesis requires further testing, but has implications for how learning and valuation may occur in more complex tasks and situations.

Brain regions correlating with EV signals also overlapped with the network of regions that have been shown to encode subjective value; and indeed some of these regions are the same as those in the core retrieval network discussed above^37^,^38^,^39^. In particular, overlapping areas in medial PFC and precuneus correlated with unique signals from both MS-EV and ERO-EV. A recent economic decision-making study showed a similar pattern of results: both value and subjective confidence were separately identifiable in overlapping areas of ventromedial PFC and precuneus^38^. This study also found that subjective confidence, but not value, was represented in a more anterior region in right rostrolateral prefrontal cortex. We similarly observed activation for ERO-EV, but not MS-EV, in more anterior PFC. This suggests that the anterior portions of PFC may represent metacognitive confidence signals, but not value signals, in both economic and mnemonic decisions.

Future work will need to address the functional form of the MS-EV signal. There are several plausible hypotheses regarding this signal: first, the EV pattern may be task-independent such that individuals have learned, in a Pavlovian manner, that higher MS and successful retrieval is generally associated with positive outcomes across many tasks. Second, the EV may be task-dependent: because the current task is framed as a memory test, participants may assign higher value to high-MS states, since high MS is more likely to be associated with the explicit (or assumed) goals of the memory test (that is, remembering old items)^19^. Third, because the calibration between confidence and accuracy is typically better for old than for new responses in recognition memory, the better calibration associated with old responses (corresponding to higher MS) may be valued by individuals during the task^52^.

Furthermore, the underlying source of the MS-EV signal in the brain remains to be specified. There are separate oldness and novelty inputs into the memory-decision system, and the EV signal may be derived either from the oldness signal or from an integration of both signals^53^,^54^. A related model has been used in the context of perceptual decisions^55^. Alternatively, the EV signal could itself represent some form of PE that is scaled relative to a prospective expectation regarding the retrieval attempt. For example, the value of a retrieval attempt (during the presentation of the test item) may itself be scaled relative to a prospective metacognitive judgment about the likelihood of remembering that particular item (for example, akin to a Judgment of Learning).

Striatal activity also is likely modulated by a variety of value-related factors related to the content of the retrieved information, such as the affective or emotional content of retrieved memories^56^, and external incentives^19^,^20^. Importantly, however, the memoranda in the current task did not have an explicit or systematic affective component, and successful retrieval activates striatum even in the absence of task feedback^19^,^36^. This suggests that the striatal value signal we observe here occurs in an obligatory or automatic fashion during retrieval and is not necessarily dependent on the content of the memory or other external factors. In addition to supporting the task-general control that is the focus of the current study, this value signal might also support adaptive re-encoding of specific retrieved information^18^. During encoding, engagement of the nigra-striatal system by reward, expectations, and active choice supports enhanced long-term memory^46^,^57^. During retrieval, the striatum may index behaviourally relevant information and facilitate re-encoding of the information for future retrieval^18^.

We emphasize that the present results do not rule out a complementary role of the ERO signals on recognition decisions. We observed ERO-PE signal in right inferior frontal gyrus and bilateral inferior parietal cortex. Previous studies have identified PEs associated with surprising state transitions in lateral prefrontal and inferior parietal cortex^16^. The ERO-PE signal may reflect participants' surprise when feedback deviates from metacognitive expectations^6^, for example, positive feedback following a low confidence response. Additional work is needed to explore the potential role of this signal in declarative memory.

The present work not only informs the field of declarative memory research but also provides a new domain in which to test RL theory. There is a growing understanding of the contributions of striatum and RL to higher-level cognition^12^,^15^,^16^,^17^, and the dynamics of RL in declarative memory may differ in important ways from typical RL tasks. Many RL tasks involve learning simple response-outcome mappings for concrete stimuli, although these principles have been extended to more complex actions and policies^16^,^17^. Our results suggest that RL occurring during recognition memory is not at the level of a simple stimulus–response-outcome mapping. Instead, the modelling results and successful transfer to free recall suggest that the RL mechanisms update an evaluative control process that operates on the latent and multidimensional contents of retrieved memory representations. That is, RL principles generalize beyond overt actions and action policies to cognitive actions like memory retrieval. Highlighting the importance of explicitly testing RL predictions in other cognitive domains, we found that MS-PE rather than ERO-PE drove striatal responses and learning in the current experiments. This shows that value can derive from complex latent processes like retrieved memory, instead of (or in addition to) the overt outcome of a plan or response. This motivates future work that investigates the generality of how RL mechanisms regulate cognition.

The current study provides a clear motivation to test causal striatal contributions to declarative memory in patients with nigra–striatal dysfunction, such as Parkinson's or Huntington's disease. Although these patient populations show profound impairments in skill learning^58^, they also show subtler deficits in declarative memory retrieval similar to the deficits seen in patients with frontal lobe damage, and this pattern may be due to their inability to appropriately evaluate and update retrieval policies^18^. Broadly, our findings suggest that successful control of declarative memory retrieval can be considered a skill that is learned and updated through the nondeclarative reinforcement learning system. This perspective may have important implications for memory training interventions in clinical and educational settings.

## Methods

### Participants in fMRI experiment

Nineteen right-handed adults (age=18–29 years, mean=22.8 years; 7 female) with normal or corrected-to-normal vision completed the neuroimaging study and are included in the analyses. All participants were without psychiatric or neurological conditions, contraindications for MRI, or medications affecting the central nervous system. All participants gave written informed consent and were compensated $20 per hour according to guidelines established and approved by the Institutional Review Board of the Research Protections Office at Brown University. Four additional participants completed the experiment but were excluded *a priori* from all analyses because of excessive head movement detected during preprocessing (>3 mm translation in a single run). We did not predetermine sample size, but our sample size is within the range used in previous behavioural studies of this paradigm^28^ as well as recent fMRI studies of memory from our lab^6^,^7^ and others^19^,^20^,^56^,^57^.

### Task procedure and analyses for fMRI experiment

The stimulus set consisted of 640 nouns naming concrete objects. For each participant, 280 words were randomly selected to be used in the study phase, 240 of which were included as studied items in the test phase. An additional 240 words were randomly selected to be used as unstudied (lure) items in the test phase.

The study task took place on a laptop computer outside the scanner. Participants were instructed to make one of two semantic judgments about each presented item and were not forewarned of the upcoming recognition test. On each trial, a word was presented for 300 ms, followed by a fixation screen for 1 s, followed by the response prompt. The prompt indicated which semantic judgment to make by providing the possible answer choices: organic/inorganic or small/large (relative to a typical-sized shoebox). Participants had 1 s to indicate their response with a key press and failure to respond on time elicited an auditory tone as feedback; no other feedback was provided for any responses. Participants completed 280 trials with an inter-trial interval of 1.2 s. Self-paced breaks were provided after the 96th and 192nd trials. The order of the study words was randomized for each participant. These study tasks were chosen because prior studies from our lab^7^ have found that combining these tasks maintains participants' engagement with the experiment and result in good recognition performance even when participants are not forewarned of the memory test. The two study tasks resulted in a small difference in hit rates during recognition (hit rate for items studied with organic judgement: 82.9%; size judgement: 85.8%, *P*<0.05). Because we did not have any *a priori* predictions regarding how this factor might interact with feedback, we did not analyse this factor further and it was not included in any of the analyses of the imaging data.

The recognition test was completed within the MRI scanner. The test phase consisted of 480 trials divided into six runs each seven minutes and ten seconds in length; participants were allowed a self-paced break between runs. Participants were told that they would be presented with a mix of studied (old) and unstudied (new) items and to respond to the test prompt ‘Is this item old?' by indicating ‘YES' with their index finger and ‘NO' with their middle finger on the response box (Fig. 2a). We refer to ‘YES' responses as old responses and ‘NO' responses as new responses throughout the manuscript. Participants had 1.5 s to initiate their response from the time the test item appeared on the screen; response time was coded based on the elapsed time between item presentation and the initiation of the button response.

Participants were further instructed to indicate their confidence by holding down the response button. This caused a bar to move along an unfilled rectangle underneath the corresponding ‘YES' or ‘NO' response (Fig. 2a). Participants were told that the top of the rectangle corresponded to high confidence and the bottom of the rectangle corresponded to low confidence. They were told to position the bar to match their confidence and encouraged to use the whole range of confidence ratings for their responses. The bar moved at a speed such that it took 1.5 s to move across the entire rectangle, which was 300 pixels high. Confidence on each trial was scored from 0 to 1 based on the position of the bar within the rectangle. To deconfound confidence rating from the movement of the bar, the bars started at the top of the rectangles on half of trials and the bottom of the rectangles on the other half. This positioning was always visible to the participant before they initiated their response. If participants wanted to indicate a confidence rating that corresponded to the end of the rectangle where the bar started, they were told to press the response button very briefly (for example, a low confidence response when the bar starts at the bottom of the rectangle). The test prompt, probe word, and answer choices remained on screen for the duration of the confidence rating.

A fixation screen of variable duration followed the response/confidence rating and was followed by a feedback screen indicating either ‘Correct!' or ‘Incorrect!' (Fig. 2a). False positive feedback was provided following approximately 70% of incorrect old responses to new items (false alarms). For each participant, the experimental script tracked the range of confidence responses provided for false alarms to determine the median false alarm confidence rating before each trial. Hundred per cent of false alarms made with a confidence rating below this median received false-positive feedback, and ∼40% of false alarms made with a confidence rating above this median received false-positive feedback. All other trials received veridical feedback.

False feedback was targeted to low confidence false alarms because both the MS-PE and ERO-PE alternatives predict that this pattern of feedback will result in a large behavioural effect: a more liberal criterion (Fig. 1). Participants received an average of eight false feedback trials per block. After feedback, a filler fixation screen was presented such that the total length of the trial, not counting the variable inter-stimulus interval between response and feedback, summed to 4 s. This filler fixation screen transitioned seamlessly to the inter-trial interval fixation screen of variable duration.

Design efficiency was optimized using a combination of Optseq2 (http://surfer.nmr.mgh.harvard.edu/optseq/) and custom-made scripts. An equal number of studied and unstudied items were presented in each run; the probe word presented on each studied/unstudied trial was randomly drawn from the studied and unstudied items for each participant. The order of studied and unstudied items, the timing of the inter-trial interval, and the timing of the inter-stimulus interval were optimized to maximize detection power and allow us to separably estimate effects associated with response from those associated with feedback. The duration of the inter-trial interval fixation had a mean of 2 s and a range of 1–11 s. The duration of the inter-stimulus fixation between response and feedback had a mean of 2.85 s and a range of 1–6 s.

To acquaint participants with the response method used for the recognition test, they completed an ostensibly unrelated estimation task immediately following the study phase and before the test phase. The display was visually similar to the display used in the recognition test, but the test word was replaced with either ‘right' or ‘left' and a number between 0 and 100. Participants responded with either the left or right arrow key on the laptop and moved the bar to the corresponding position in the appropriate rectangle. They completed 40 trials of this practice task. Feedback was only provided on non-response trials.

We computed the recognition criterion for each run (80 trials) of the scanning session using the standard signal-detection approach^59^. Criterion was calculated as:


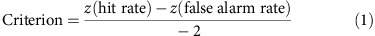


Initial criterion refers to criterion for the first run and terminal criterion refers to criterion for the final run. Criterion values from all runs were submitted to a repeated-measures ANOVA with Run as a factor, and follow-up *t*-tests as described in the Results. Note that we use the terminal criterion values, instead of a difference score between terminal and initial criterion, for our analyses because false-positive feedback occurred immediately from the onset of the experiment, and thus initial criterion does not provide a clean baseline to compute a difference score. See Supplementary Note 1 and Supplementary Fig. 4 for additional discussion of this metric.

### fMRI data collection and preprocessing

Whole-brain imaging was performed on a Siemens 3T TIM Trio MRI system. High-resolution T1-weighted (multi-echo MP-RAGE) anatomical images were acquired for visualization (TR=2200, ms; echo times=1.54 ms, 3.36 ms, 5.18 ms, 7.00 ms; flip angle=7°; 144 sagittal slices; 1.2 × 1.2 × 1.2 mm). Functional images were collected over six runs using a gradient-echo echo-planar sequence (TR=2 s; echo times=28.0 ms; flip angle=90°; 40 axial slices; 3 × 3 × 3 mm). Head motion was restricted using firm padding surrounding the head. Stimuli were projected onto a screen and viewed through a mirror attached to a 32-channel head coil. Responses were provided with the right hand through a Mag Design and Engineering MRI-compatible four-button response pad. The first five volumes of each functional run were discarded to allow for T1 stabilization.

Preprocessing and data analysis were performed using SPM8 (www.fil.ion.ucl.ac.uk/spm). Image data quality was first assured via visual inspection and using customized versions of TSDiffAna (http://sourceforge.net/projects/spmtools/) and ArtRepair software (http://cibsr.stanford.edu/tools/human-brain-project/artrepair-software.html). Functional images were then corrected for differences in slice acquisition timing by resampling all slices to match the first slice. Images were then motion corrected across all runs using B-Spline interpolation. For four participants that had head motion >3 mm across the entire session but not within a single run, motion-correction was applied separately for each run. As already noted, participants with movement of >3 mm within a run (*N*=4) were excluded. Nuisance regressors were included for all participants to account for run-to-run variance. Data were then normalized based on MNI stereotaxic space and then spatially smoothed with an 8 mm FWHM isotropic Gaussian kernel.

### fMRI data analysis and visualization

Data analysis was conducted under the assumptions of the general linear model as implemented in SPM8. Single subject effects were estimated using a fixed-effect model. All condition regressors were generated by convolving stick functions (duration=0) with a canonical hemodynamic response function and its temporal derivative. Nuisance regressors were included to account for run-to-run variance and low-frequency signal components. Linear contrasts at each voxel were used to obtain subject-specific estimates for each effect. These estimates were entered into second-level analyses treating subjects as a random effect, using a one-sample *t*-test against a contrast value of 0 at each voxel.

Activations detected with whole-brain analyses were considered statistically reliable to the extent that they survived a false discovery rate (FDR)-corrected threshold of *P*<0.05 at the cluster level. Whole-brain maps were initially thresholded at *P*<0.001, uncorrected, and cluster corrected to *P*<0.05 using SPM's FDR algorithm. The critical cluster extent for each contrast is listed in each corresponding table.

All Figures are from group contrasts rendered on the MNI canonical brain in neurological convention. The statistical thresholds used for display purposes are listed in the Figure captions. All coordinates are in MNI space and correspond to the peak voxel in the cluster. Local maxima reported by SPM within a cluster are also reported. Microanatomical labels were determined based on inspection of the Talairach and Tournoux atlas^60^, and macroanatomical labels were determined using the Harvard-Oxford Probabilistic Atlas implemented in FSL (www.fmrib.ox.ac.uk/fsl) and prior literature.

Whole-brain analyses were complemented by Region of Interest (ROI) analyses. ROIs were defined using anatomical masks from the Automated Anatomical Labeling (AAL) database^61^. Our striatum ROI was constructed by combining the AAL definitions of bilateral caudate and putamen (total volume: 32,232 mm^3^), as our lab has used previously^6^. We also constructed an anatomical ROI guided by the functional activations associated with our ERO-PE regressor, to test for an ROI × effect interaction, as described in the Results. This ROI was constructed by combining the AAL definitions of bilateral inferior parietal cortex and right inferior frontal gyrus opercularis (total volume: 41,528 mm^3^). The rationale for selecting this second anatomical ROI was simply to test the statistical reliability of the qualitative interaction pattern (between MS-PE and ERO-PE) seen in the whole-brain analyses. Whereas MS-PE was associated with activity in striatum but not bilateral parietal and right IFG, ERO-PE was associated with bilateral parietal and right IFG, but not striatum. However, these separate effects from the whole-brain GLMs are inadequate to demonstrate the region × effect interaction^34^,^62^, necessitating an ROI approach. To assess ROI activation, the time-series signal averaged across the entire ROI was extracted using the MarsBaR toolbox^63^, and the respective GLM was regressed against this time-series. The parameter estimates (beta values) associated with each regressor were computed and the parameter estimates for regressors of interest were used subjected to analysis of variance, *t*-tests, and correlation analyses as described in the Results. All reported *P*-values are two-tailed unless explicitly noted otherwise, and considered significant at *P*<0.05 for tests of behaviour and brain-behaviour correlations. All group averages are mean values. All error bars indicate s.e.m. Across all experiments, the normality of the behavioural data was confirmed using Shapiro–Wilk tests and differences in variance were assessed using either Leven's tests (*t*-tests) or Mauchly's tests (ANOVA). In the case of violations of sphericity, Greenhouse–Geisser corrected degrees of freedom and *P* values are reported.

### Calculating expected value and prediction error values

Parametric regressors for fMRI analyses were calculated using trial-by-trial EVs and PEs under both the MS and ERO alternatives. Our approach is intended to frame recognition memory decisions in the same framework as value-based decision-making models, and we sought to adopt the conventions of these models. On the basis of typical RL models, positive outcomes in our calculations correspond to 1 (presence of rewarding outcome, or ‘Correct!' feedback) and negative outcomes correspond to 0 (absence of rewarding outcome, or ‘Incorrect!' feedback). Similarly, the EV associated with a decision is also scaled from 0 (low expectation of a positive outcome) to 1 (high expectation of a positive outcome). PE, then, is defined as the difference between the EV and the outcome (1 or 0) and thus ranges from −1 to 1. We calculated a MS-EV, MS-PE, ERO-EV and ERO-PE) for all trials on which a participant made a response.

For the MS alternative, we transformed response confidence on each trial to derive a MS-EV as depicted in Fig. 1, scaled from 0 for the lowest MS (high confidence new responses) to 1 for the highest MS (high confidence old responses). For new responses, maximum confidence corresponded to an MS-EV of 0 and minimum confidence corresponded to an MS-EV of 0.5. That is, MS-EV for trials with a new response was calculated as:


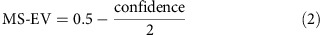


For old responses, minimum confidence corresponded to an MS-EV of 0.5 and maximum confidence corresponded to an MS-EV of 1. That is, MS-EV for trials with an old response was calculated as:


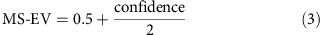


Importantly, this coding for the MS alternative allows for the MS of both old and new items to vary from 0 to 1, but constrains the MS associated with new decisions to 0 to 0.5 and with old decisions to 0.5 to 1. Finally, for each trial, we calculated the MS-PE by taking the difference between the presented outcome (veridical and false positive feedback: 1; negative feedback: 0) and the calculated MS-EV for that trial:


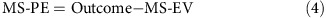


For the ERO alternative, we derived an ERO EV (ERO-EV) on each trial that was directly related to response confidence: for both new and old responses, minimum confidence responses corresponded to an ERO-EV of 0 and maximum confidence responses corresponded to an ERO-EV of 1, as depicted in Fig. 1. That is, ERO-EV on all trials was calculated as:


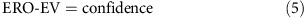


For each trial, we calculated the ERO-PE by taking the differences between the presented outcome and ERO-EV for that trial:


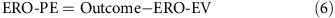


### fMRI general linear models

We first constructed two versions of our GLM: a MS GLM and an ERO GLM. The GLMs were identical except for the parametric regressor that was included. We included a condition regressor for trial onset and a condition regressor for the presentation of feedback. In each GLM, the trial onset regressor was modulated by the respective EV parametric regressor and the feedback presentation regressor was modulated by the respective PE parametric regressor. These parametric regressors were convolved with the HRF and temporal derivative basis functions. We further included separate condition regressors for the trial onset and feedback events of non-response trials on which the participant made an error of omission. These regressors were treated as nuisance regressors; they were not modulated by a parametric regressor and were not analysed. We included identical nuisance regressors in our third and fourth GLMs described below.

In each of these models the parametric regressor captures all variance associated with that regressor above and beyond the variance captured by the primary condition regressor. For example, the MS-PE regressor in the MS GLM would include only variance above and beyond the main effect of feedback presentation, and would include variance shared with the ERO-PE values as well as any variance unique to MS-PE. Likewise, the ERO-PE regressor in the ERO GLM would include variance shared with the MS-PE values and its unique variance.

The issue of correlation and shared variance between regressors is common to virtually all studies using event-related fMRI designs^64^. To mitigate this concern, the present experiment included 280 total trials and we jittered the inter-trial-interval and the delay period between the response and feedback phase of each trial to optimize design efficiency. However, because this correlation reduces the statistical power for analyses of the unique variance of MS and ERO signals, we explicitly report these correlations here. The average correlation between the MS-PE and ERO-PE predictors after convolution with the canonical HRF was *R*^2^=0.52 (the correlation was first determined within each participant, transformed with Fisher's *Z*-transformation, averaged across all participants, and transformed back to a correlation coefficient.) The average correlation between the convolved MS-EV and ERO-EV predictors was *R*^2^=0.19. Thus, we conclude that there is sufficient unique variance within each individual participant to reliably distinguish these signals in the present data.

Our third GLM included the MS-PE and ERO-PE parametric regressor in the same model. This model included a condition regressor for trial onset and a condition regressor for feedback presentation; these were not modulated by any parametric regressor. Instead, we convolved the MS-PE and ERO-PE values with the HRF and input these values as two user-entered regressors. This allows the two PE regressors to compete for variance in the model and capture only unique variance^64^. This is similar to what happens when two parametric regressors are included as modulators on a condition regressor in SPM. By default, SPM performs serial orthogonalization such that the first parametric regressor includes both shared variance and its unique variance, and the second parametric regressor includes only its unique variance. For example, if a hypothetical GLM included MS-PE as the first parametric regressor and ERO-PE as a second parametric regressor, the ERO-PE regressor would only capture the unique variance. By the same logic, our fourth GLM included both MS-EV and ERO-EV parametric regressors in the same model, capturing the unique variance of each EV regressor. Like our third GLM, this model included a regressor for trial onset and a regressor for feedback presentation; these were not modulated by any parametric regressor. Instead, we convolved the MS-EV and ERO-EV values with the HRF and input these values as two user-entered regressors.

Finally, we constructed three GLMs for control analyses. The first control GLM was similar to the third GLM except that MS-EV (and thus MS-PE) was calculated differently. We refer to these values as MS-EV* and MS-PE*. Note that the MS-PE alternative predicts a main effect difference between PEs for feedback following old versus new responses. For example, PEs for positive feedback will always be larger following correct old than following correct new responses (compare orange versus blue arrows in Fig. 1c). This control GLM removed this main effect difference. Specifically, the MS-EV* and MS-PE* values were calculated as follows. For new response trials:


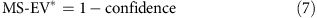


For old response trials:


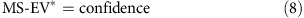


For all trials:


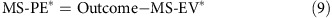


This coding thus removes the main effect difference but still captures the relative difference within old and new responses, that is, MS-EV* will increase as confidence decreases for new responses, but will increase as confidence increases for old responses. MS-PE* will show the same relative pattern across old and new responses: consider a correct new response made with high confidence (0.8) and a correct old response made with low confidence (0.2). These example responses correspond approximately to the first and third example test items shown in Fig. 1a. Under this control GLM, these responses will have the same MS-EV* (0.2) and following positive feedback would show the same MS-PE* (1–0.2=0.8). Supplementary Figure 2 shows the whole-brain activity associated with the MS-PE* regressor and reveals activity in striatum comparable to the primary MS GLM; this confirms that the striatal effect found for the unique variance of MS-PE is not solely due to the predicted main effect differences for PEs to feedback following old and new responses, but instead also captures the relative pattern of predicted PEs within old and new responses.

The second control GLM was similar to the first GLM except that the response phase and feedback phase were modeled separately for old and new response trials. This allows us to look at the PE signal (coded under the MS alternative) specifically following new responses, when the MS and ERO alternatives make categorically opposite predictions. Supplementary Fig. 2 shows the whole-brain activity associated with the MS-PE regressor and reveals activity in striatum comparable to the primary MS GLM; this test confirms that the striatal signal follows the specific pattern to feedback following new responses that is predicted by the MS alternative but not by the ERO alternative. Altogether, these control analyses provide additional support that the MS alternative better explains striatal signal.

In the final GLM, we included regressors for Hits, Correct Rejections, False Alarms, and Misses, crossed with the response phase (modeled at the trial onset) and feedback phase (modeled at the feedback presentation) of each trial. This resulted in eight total condition regressors (Hits-Response, Hits-Feedback and so on). We used this control GLM to compute the standard retrieval success (Hits-Response>Correct-Rejections-Response) contrast that is often reported in fMRI studies of recognition memory^36^, which allows for comparison between the activation in this contrast to the activation associated with the various EV and PE signals. The whole-brain results from this contrast are shown in Supplementary Fig. 2.

### Participants in behavioural recognition memory experiment

Eighty participants (age=18–27 years, mean=20 years; 45 female) participated in exchange for course credit or payment. All participants had normal or corrected-to-normal vision and were native English speakers. Participants gave written informed consent according to guidelines established and approved by the Institutional Review Board of the Research Protections Office of Brown University. After completing the experimental session, participants were thoroughly debriefed with regard to the false-feedback manipulation. Participants were randomly assigned to experimental conditions (New–High Confidence; New–Low Confidence; Old–Low Confidence; Old–High Confidence; or Veridical Feedback; *n*=16 in each group) and the experimenter running the study was blind to this assignment. The number of participants was chosen based on previous studies using a similar paradigm^28^ and a pilot experiment that did not target confidence to specific levels of confidence.

### Task procedure and analyses for behavioural experiment

The procedure for the recognition task was similar to Experiment 1 except as described here. The study phase consisted of 240 items and breaks were provided every 80 trials. After the study phase, all participants completed a probabilistic selection task in which they viewed Japanese Hiragana characters and learned to select specific characters based on trial and error^15^. None of the word stimuli used in the recognition task appeared during this task and the results from this intermediate task are not discussed further.

The test phase consisted of 288 trials equally divided among studied and unstudied items. Breaks were provided every 48 trials. The trial sequence was identical to Experiment 1 except for the visualization used to indicate confidence: instead of a thin bar moving with the unfilled rectangle (Fig. 2a, second panel), the rectangle started filling as soon as the response key was pressed and stopped as soon as the key was released. The rectangle always started filling from the bottom and the proportion of the rectangle filled indicated subjected confidence such that higher confidence was indicated by filling a larger proportion of the rectangle. To ensure that participants would make a sufficient number of errors for the false-feedback manipulation to be applied to all groups, the test phase took place ∼24 h after the study phase. Overall, participants were able to perform the task (mean proportion correct=66.53%; s.e.m.=0.77%) and accuracy values from the four targeted feedback groups were submitted to a two-way ANOVA with factors of targeted response group (Old/New) and targeted confidence (Low/High). Neither the main effects nor the interaction reached significance (all *P*>0.05).

Following from Han and Dobbins^28^ and Experiment 1, we divided our analysis of the test phase into blocks to account for the pattern of criterion over time. To approximately match the number of trials that went into each bin of Experiment 2 to the number of trials in each run of Experiment 1, we divided the test phase into thirds and calculated an initial criterion, middle criterion, and terminal criterion. Thus, each criterion calculation was based on the hit rate and false alarm rate across 96 trials (compare with 80 trials per run in Experiment 1). Because analyses of criterion were *a priori* planned tests, we considered statistical results as significant at an alpha level of *P*=0.05. Because we did not have strong *a priori* predictions about additional analyses regarding confidence, these analyses were Bonferroni corrected for multiple comparisons.

### False-feedback manipulation

Participants were assigned to one of five experimental conditions. The Veridical feedback group received completely veridical feedback. The other four conditions received false positive feedback targeted towards either low or high confidence errors. The two New groups received false-positive feedback on a subset of new errors and veridical feedback on all other trials. The false-positive feedback was targeted to either high confidence new errors (New–High Confidence) or low confidence new errors (New–Low Confidence). Two Old groups received false-positive feedback on a subset of old errors and veridical feedback on all other trials. The false-positive feedback was targeted to either high confidence old errors (Old–High Confidence) or low confidence old errors (Old–Low Confidence).

The method for targeting false-positive feedback employed an adaptive algorithm that took into account individual differences in confidence ratings and base rates of errors. This information was used to provide approximately equal instances false feedback to each participant, to provide the false feedback equally across the course of the entire recognition test phase, and to maximize the difference in the targeted confidence between subgroups. The script was designed to provide each participant ∼30 instances of false feedback. The script tracked online the participant's error rate for each response type (false alarms and misses.) For each trial in the recognition test, the experimental script estimated how many more errors the participant would make and determined the proportion of these errors that would need to receive false feedback for the participant to receive ∼30 total false-feedback instances. This proportion was updated on a trial-by-trial basis for each participant and was used to probabilistically provide false-positive feedback. See the Supplementary Methods for additional details, examples, and manipulation checks for the false-feedback algorithm.

### Computational modelling of fMRI and behavioural experiments

In the DDM, the decision process arises from noisy evidence accumulation (or drift) towards one of two response bounds^65^. For recognition decisions, one bound corresponds to an old response and the other bound corresponds to a new response. Thus, over time, evidence accumulation drifts towards one of these response bounds at a rate (that is, the drift rate; *v*) that is determined by the strength of evidence recovered from memory. Evidence that is evaluated as signalling old leads to a positive drift rate, towards the old boundary. Evidence that is evaluated as signalling new leads to a negative drift rate, towards the new boundary. The accumulation process is terminated when the drift reaches either boundary, and the corresponding response is made. Stronger evidence would lead to a larger drift rate, and thus, a faster response.

The response bias, *z*, determines the starting point of the diffusion process relative to either of the response boundaries. When bias is closer to the old response boundary, participants are overall more likely (requiring less evidence) to respond old independently of the strength of the evidence (drift rate). Shifts in response bias result in a larger effect on the leading edge of the response time distribution relative to shifts in the drift criterion^26^.

The drift criterion parameter, dc, in the DDM determines whether observation of a signal of a given strength is more likely to elicit drift towards the old or new boundary. This is not thought to change how a MS or match signal is retrieved from long-term memory (which is impacted by factors such as MS of studied items) but instead how this match signal is evaluated and mapped onto drift rate^26^,^42^,^43^,^44^,^66^. From this perspective, the drift process can be thought of as a series of small signal detection processes, where at each time step, the evidence for an item is sampled. If that sample is greater than the old/new criterion then the drift is incremented toward old, and is otherwise incremented towards new. The criterion of those signal detection processes conducted at each time step is the drift criterion. Shifts in drift criterion result in equal but opposite changes in the drift rate for each choice (that is, a higher drift rate for old or new and a slower drift rate for the other) and thus drift criterion shifts have the same impact on distributions as a symmetrical change in drift rate itself. Changes in drift criterion have a larger impact on the tail than do changes in bias^26^.

The recognition decision was modeled using the DDM, following prior work^26^,^42^,^43^,^44^,^65^,^66^. For all model variants, parameters were fit to the accuracy and response time data (from both correct and error trials). In all models, we estimated parameters for an initial non-decision interval (*t*), thought to reflect processes like encoding of the target; and the distance between the boundaries (*a*); and drift rate (*v*). Thus, both models had the same number of parameters. The models differed in they each included an additional parameter, which was allowed to vary from run to run. In the Drift Criterion model, we estimated a parameter for drift criterion, which was allowed to vary from run to run. In the Response Bias model, we estimated a response bias parameter which was allowed to vary from run to run. Response time data were examined for outliers: any response faster than 300 ms was considered a guess or an error and excluded from the analyses^41^,^43^. Non-response errors (when participants failed the response deadline) were excluded from analysis. These constituted fewer than 5% of trials on average across participants.

Model fitting was conducted using the Hierarchical Drift Diffusion Modeling (HDDM)^40^ module with stimulus coding (http://ski.clps.brown.edu/hddm_docs/index.html). This Python module uses a hierarchical Bayesian estimation procedure that fits the DDM parameters based on all participant data simultaneously. This is analogous to random effects estimation in that it treats between-subject variance as a random variable, while fitting within-subject parameters simultaneously. For example, each model produces an initial non-decision interval (*t*) parameter estimate for each participant that is constrained by the group distribution for that parameter but is allowed to vary from the group distribution to the extent that the participant's data is sufficiently diagnostic^40^. A Markov-chain Monte-Carlo (MCMC) procedure estimated the DDM parameters' posterior distributions. 10,000 samples from the distributions were estimated. The first 3,000 samples were discarded (burn in), and of the remaining samples, every tenth sample was retained (thinning). Model convergence was assessed based on Monte-Carlo error (MC error) and visual assessments of chain convergence^67^. Model selection was based on minimization of the deviance information criterion (DIC), which is more readily compatible with MCMC estimation than Akaike information criterion or Bayesian information criterion. Although model fit statistics were used to select the best-fitting model, it is important to confirm that the best-fitting model adequately reproduces the key behavioural patterns it is intended to capture^40^. Supplementary Figure 7 shows posterior predictive checks, which compare the data simulated from each model to the empirical data and demonstrates that the response time distributions and changes in behavioural criterion are captured by the DDM models. We note that the model comparison between the Drift Criterion and Response Bias model is not meant to imply that there is no plausible role for changes in response bias in regulating recognition decisions; indeed, the posterior predictive checks reveal that the Response Bias model provides credible estimates of key patterns in the behavioural data. Instead, the model comparison is meant to test which parameter best captures the behavioural data and simply implies a more prominent role for changes in drift criterion in the regulation of recognition decisions.

The computational modelling for Experiment 2 was similar but included the additional factor of the experimental condition (Supplementary Fig. 6). In the Drift Criterion model, the drift criterion parameter was allowed to vary as a function of epoch (initial/middle/terminal) and each individual's drift criterion estimate was constrained by the distribution of their respective group (Veridical; New–High Confidence; New–Low Confidence; Old–Low Confidence; Old–High Confidence). Likewise, in the Response Bias model, the response bias parameter was allowed to vary as a function of epoch and each individual's response bias estimate was constrained by the distribution of their respective group.

In our implementation of the HDDM model, liberal values of *z* and dc correspond to positive values. However, in signal-detection theory, liberal values of criterion correspond to negative values (liberal values are plotted down). Therefore, to facilitate visual comparison between the HDDM results and signal-detection results in Fig. 4, we plot −dc in Fig. 4b so that liberal values correspond to negative values in both panels.

### Participants in free recall experiment

Forty-six participants (age=18–28 years, mean=20.2 years; 28 female) who did not participate in the fMRI experiment participated in the recognition-free recall experiment. Five participants were excluded from all analyses because they indicated on a post-test questionnaire that they were familiar with the DRM paradigm, based on similar exclusion criterion in previous studies that used similar paradigms^45^. All participants gave written informed consent and were compensated $10/hour according to guidelines established and approved by the Institutional Review Board of the Research Protections Office at Brown University. Participants were randomly assigned to a condition and experimenters were blind to the learning condition assignment of each participant during the experimental session and during coding of free recall responses. The number of participants was chosen based on previous studies that used similar paradigms^45^. We note that a subset of the participants included in this experiment and analysis are also included in the behavioural analysis of recognition memory criterion reported for Experiment 2: 10 of Biased Feedback–Liberal participants and nine of the Biased Feedback–Conservative participants are included in the Old–Low Confidence and New–High Confidence groups, respectively.

### Task procedure and analyses for free recall experiment

Participants first completed a false-feedback recognition paradigm identical to the one used for Experiment 2. One group, termed the Biased Feedback–Liberal group, received false feedback corresponding to the Old–Low Confidence condition of Experiment 2, chosen to maximize the effectiveness of the liberal criterion shift. The second group, termed the Biased Feedback–Conservative group, received false feedback corresponding to the New–High Confidence condition of Experiment 2, chosen to maximize the effectiveness of the conservative criterion shift.

Immediately following the recognition test, participants completed study/test cycles for ten DRM lists^68^. The 10 lists with the highest rate of free recall critical lure intrusions from Stadler, Roediger, and McDermott^69^ were selected. Within each list, the words were presented in the order they are listed in Stadler, Roediger, and McDermott^69^. Eight permutations of list order were created and each participant received one of these orders at random. The fifteen study words were presented one at a time on the screen and the participant read the word aloud at a rate of one per 2 s. Immediately following study, a prompt initiated the recall phase and participants had 2 min to verbally recall as many words as they ‘were reasonably sure had been presented on the list'.

Raters blind to the experimental condition listened to the recall verbalizations of each participant using the Penn TotalRecall toolbox (http://memory.psych.upenn.edu/TotalRecall) and coded for the presence or absence of the critical lure on each list. The total number of critical lures recalled by each participant was calculated and submitted to *t*-test and correlation analyses. On the basis of previous results^45^, we predicted that the Biased Feedback–Liberal group would recall more critical lures than the Biased Feedback–Conservative group. Thus, we performed a directional *t*-test and report the one-tailed *P* value. For the correlation analysis, we computed the recognition criterion for initial, middle and terminal thirds of the experiment and used the Terminal Criterion for the correlation with critical lure recall. We focused our analysis on critical lure recall, instead of correct recall of studied items, because previous work demonstrated a link between trait recognition criterion and critical lure recall but not correct recall^45^. We note that terminal criterion showed a modest but not statistically reliable correlation with correct recall (*R*=−0.30; *P*=.06). Thus, although criterion shifts likely influence the evaluation of true memories, it is likely that shifts in criterion simply have a larger and more detectable impact on more ambiguous memories like false alarms induced by the DRM procedure.

### Data availability

Data from all experiments are available from the corresponding author on request.

## Additional information

**How to cite this article:** Scimeca, J. M. *et al*. Striatal prediction errors support dynamic control of declarative memory decisions. *Nat. Commun.* **7,** 13061 doi: 10.1038/ncomms13061 (2016).

## Supplementary Material

Supplementary Information Supplementary Figures 1-8, Supplementary Tables 1-4, Supplementary Note 1 and Supplementary Methods
