# Supplementary material for: Striatal prediction errors support dynamic control of declarative memory decisions
Source: Nat Commun. 2016 Oct 7;7:13061. doi: 10.1038/ncomms13061 (PMC5059768; doi:10.1038/ncomms13061)
Supplement: Supplementary Information — Supplementary Figures 1-8, Supplementary Tables 1-4, Supplementary Note 1 and Supplementary Methods [file ncomms13061-s1.pdf]

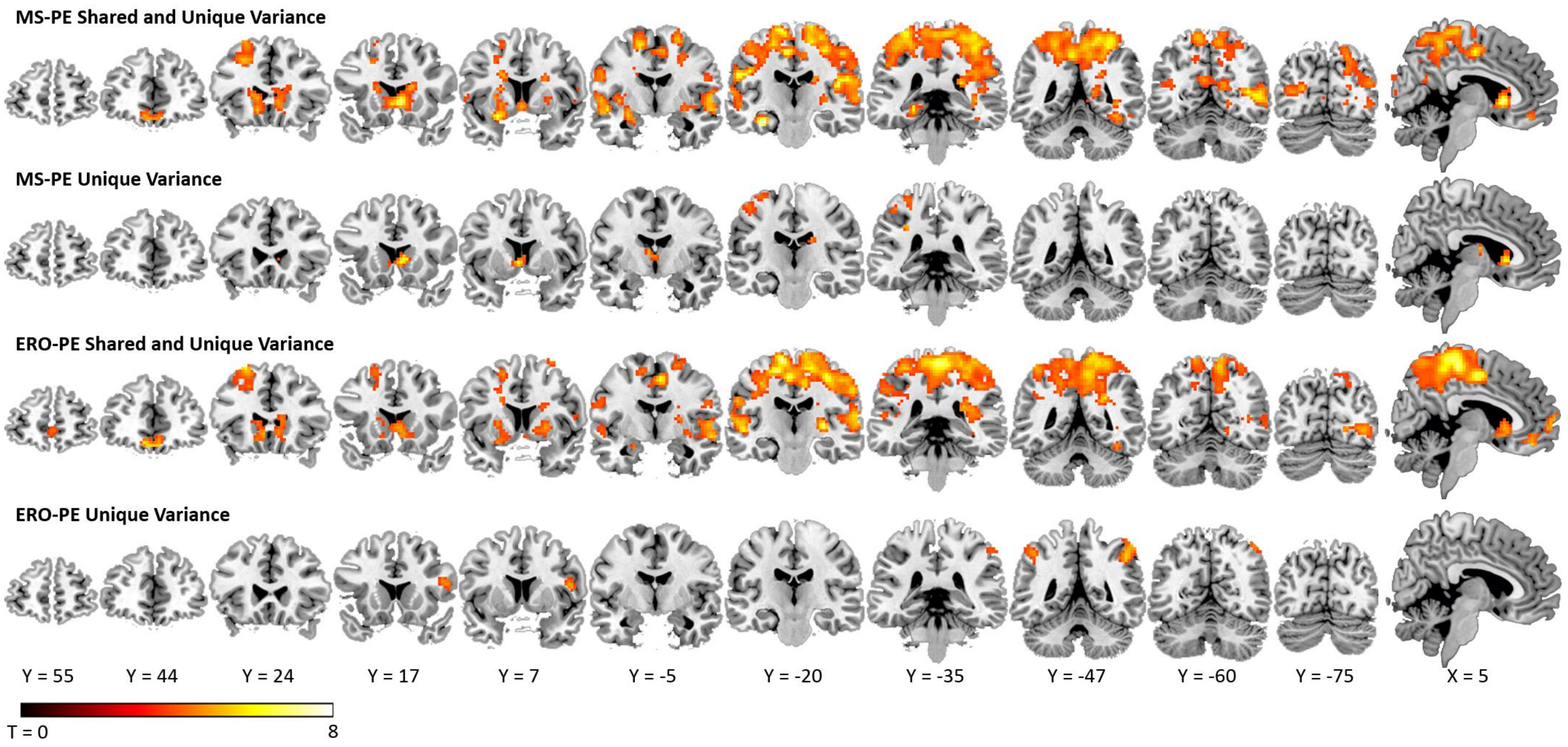

Supplementary Figure 1. Whole brain activation maps showing correlates of Prediction Error signals. The first row shows the shared and unique variance associated with the Memory Strength PE (MS-PE); the second row shows just unique variance associated with MS-PE. The third row shows the shared and unique variance associated with the Expected Response Outcome PE (ERO-PE); the fourth row shows just the unique variance associated with ERO-PE. Whole brain statistical maps are thresholded at $p < .05$, FDR cluster corrected. Color maps represent t statistics with a range from 0 to 8.

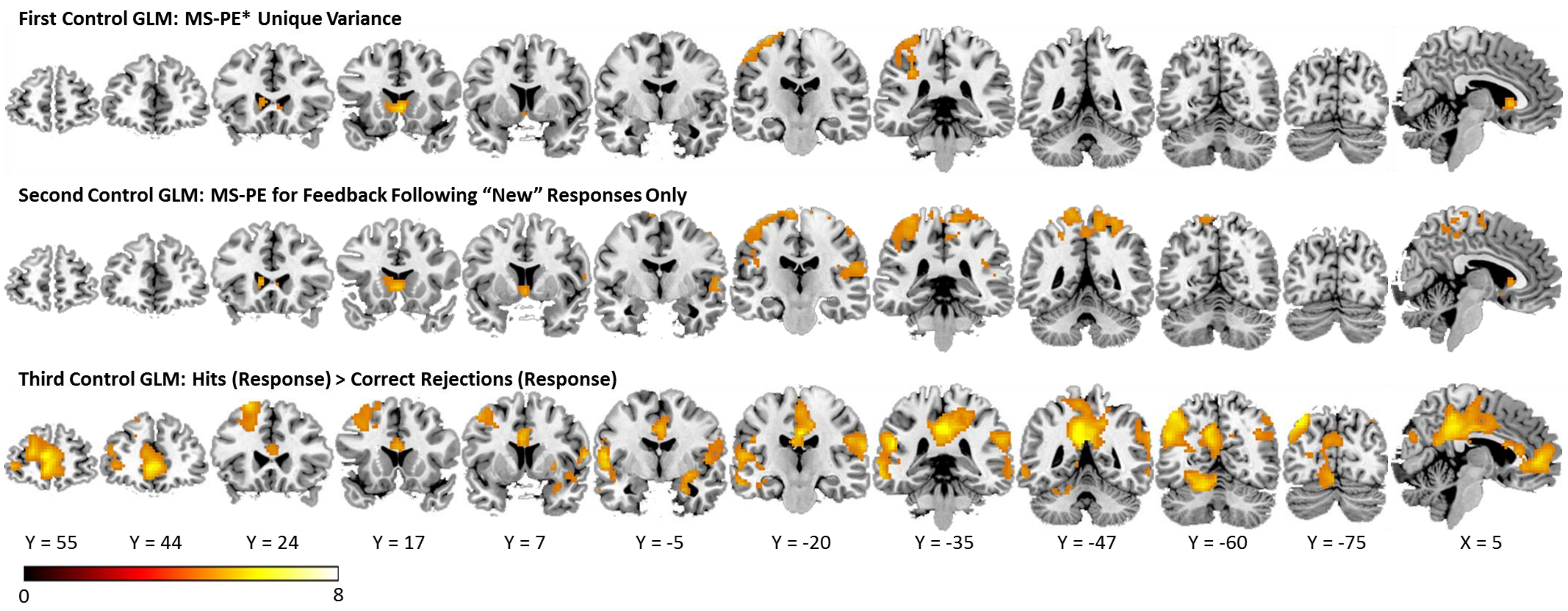

Supplementary Figure 2. Whole brain activation maps showing results from control General Linear Models. The first row shows the unique variance associated with the Memory Strength Prediction Error after removing the main effect difference for feedback following *old* versus *new* responses (MS-PE*). This analysis reveals activation in striatum that is statistically significant to but less extensive than the primary analysis of MS-PE that includes the predicted main effect. The second row shows the MS-PE signal following feedback to *new* responses, when the MS and ERO alternatives make categorically opposite predictions. This analysis also reveals activation in striatum that is statistically significant but less extensive than the primary analysis of MS-PE that includes all trials. The bottom row shows activation associated with the standard retrieval success contrast (Hits > Correct Rejections, modeled during the Response phase of the trial), which allows for comparison between the activation in this contrast to the activation associated with the various EV and PE signals. As expected, the pattern of activation is similar to the Expected Value analyses reported in the main manuscript and generally consistent with previous reports of the retrieval success contrast. The pattern found for this contrast is distinct from the MS-PE and ERO-PE contrasts. There is not reliable activation in the ventral striatum. Unlike the ERO-PE analysis, this contrast does not show reliable activation in right prefrontal cortex. The Hits > Correct Rejection contrast is associated with activation in parietal cortex, although in more posterior and ventral regions than in the ERO-PE analysis.

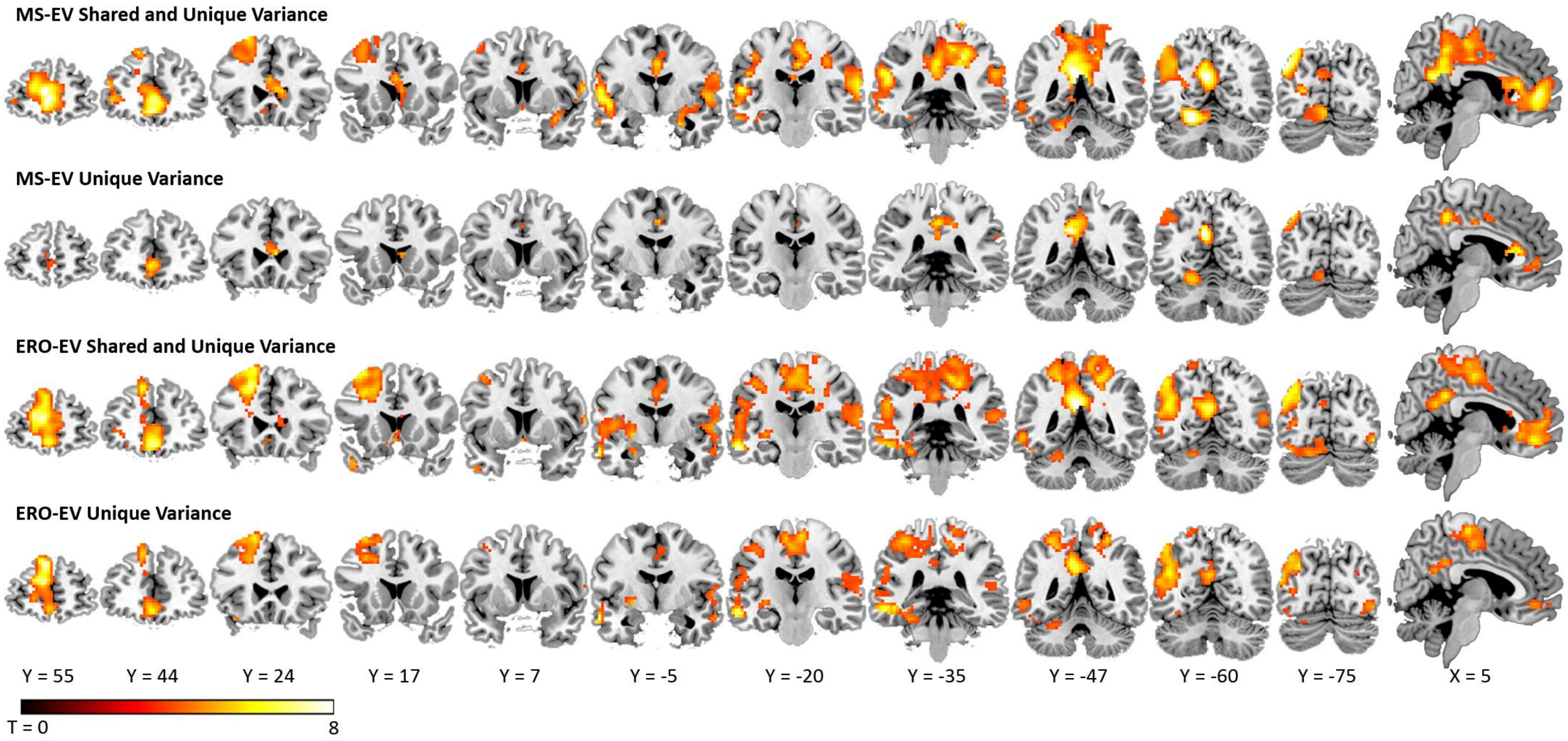

Supplementary Figure 3. Whole brain activation maps showing correlates of Expected Value signals. The first row shows the shared and unique variance associated with the Memory Strength EV (MS-EV); the second row shows just unique variance associated with MS-EV. The third row shows the shared and unique variance associated with the Expected Response Outcome EV (ERO-EV); the fourth row shows just the unique variance associated with ERO-EV. Whole brain statistical maps are thresholded at $p < .05$, FDR cluster corrected. Color maps represent t statistics with a range from 0 to 8.

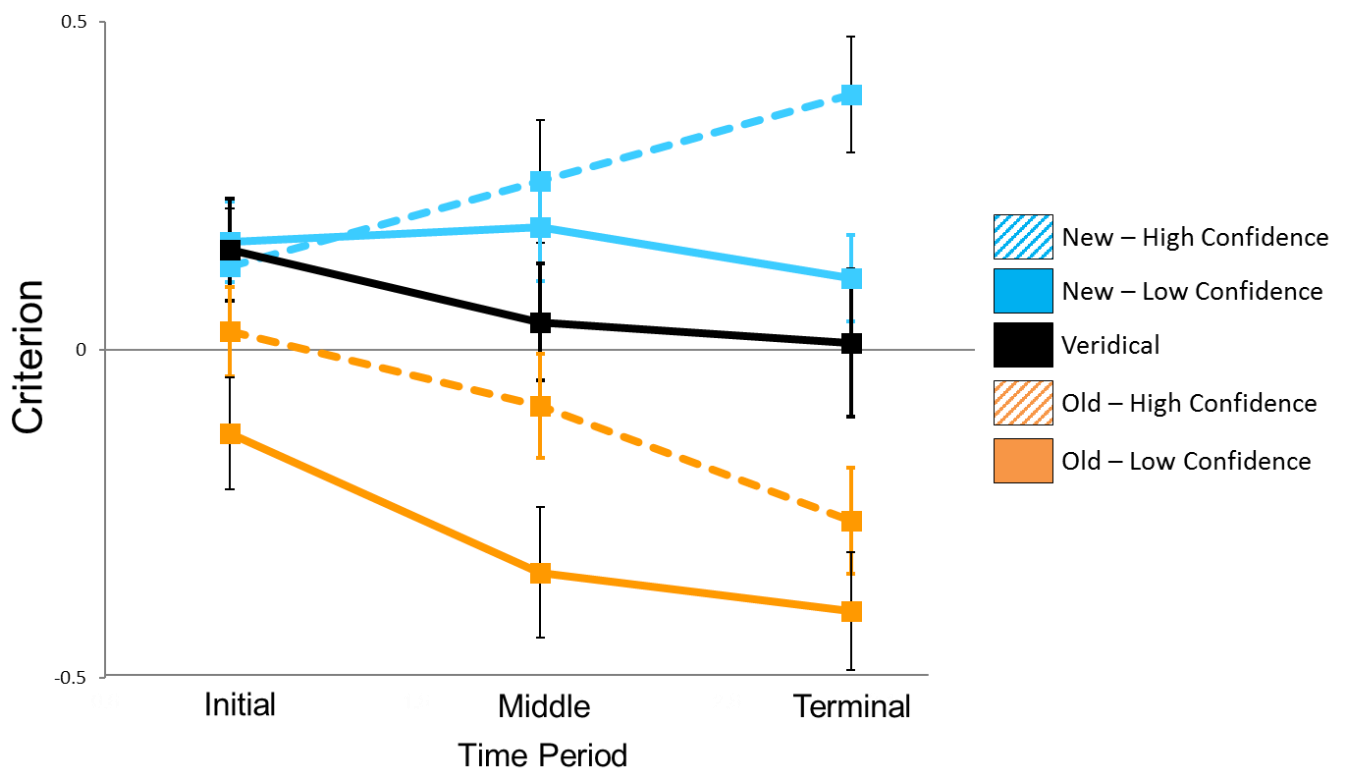

Supplementary Figure 4. **Criterion as a function of time in Experiment 2**. Signal detection estimates of criterion across experimental groups over the initial, middle, and terminal time periods of the of the recognition test session in Experiment 2. Error bars indicate s.e.m. Liberal criterion values correspond to negative values on the vertical axis.

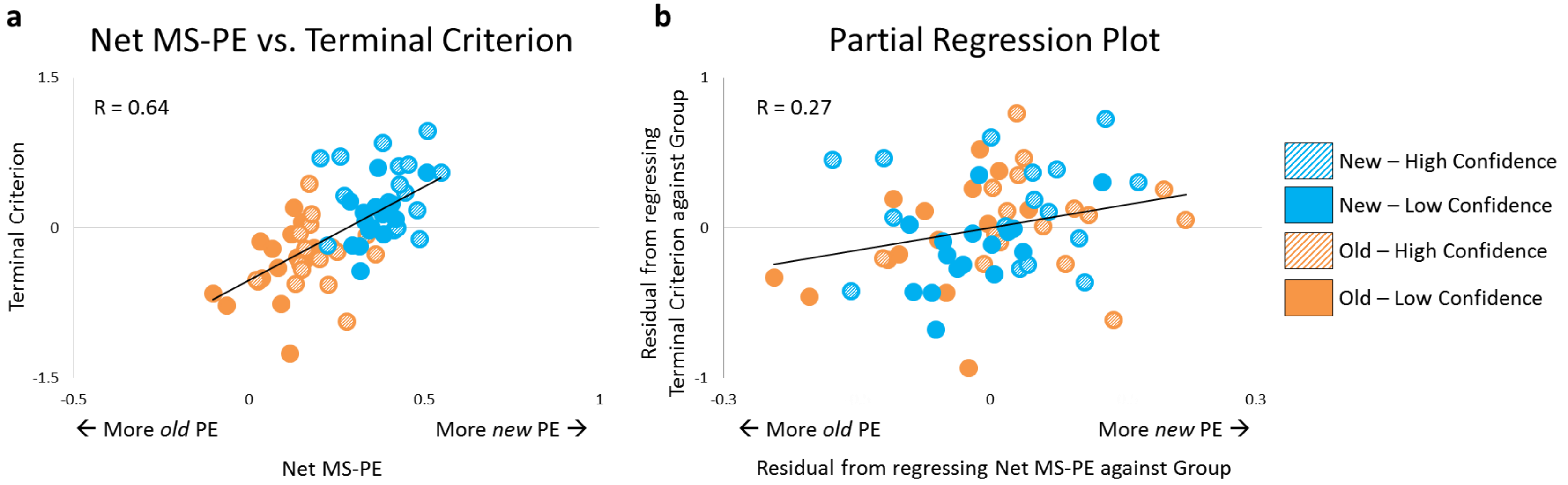

Supplementary Figure 5. Individual differences analysis relating Net MS-PE to Terminal Criterion in Experiment 2. (a) Scatterplot and best fit line showing the correlation between Net Memory Strength – Prediction Error (MS-PE) and Terminal Criterion. The color and pattern indicates the experimental condition of individual data points. (b) Partial regression plot showing the reliable relationship between Net MS-PE and Terminal Criterion after controlling for the effect of targeted response group.

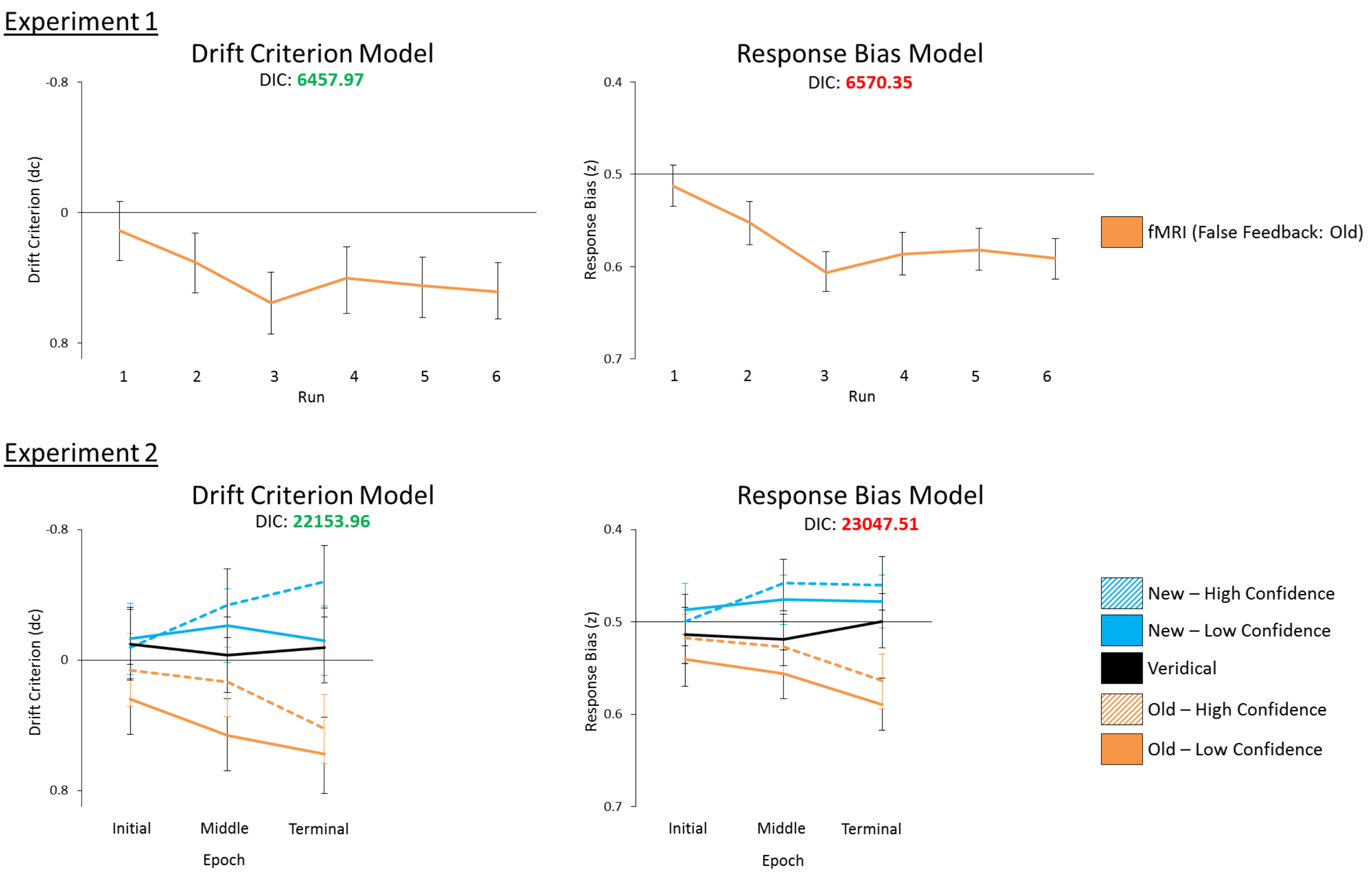

Supplementary Figure 6. DDM drift criterion and response bias estimates as a function of time. The left column plots the drift criterion (dc) values as a function of time derived from the Drift Criterion models. The right column plots the response bias (z) values derived from the Response Bias models. More liberal values are plotted down. The top row is from Experiment 1 and the bottom row is from Experiment 2. The DIC (deviance information criterion) values were used for model-selection; the preferred model is the one with a lower DIC. As a general heuristic, differences in DIC larger than about 10 constitute meaningful differences in model-fit. Error bars represent 95% confidence intervals.

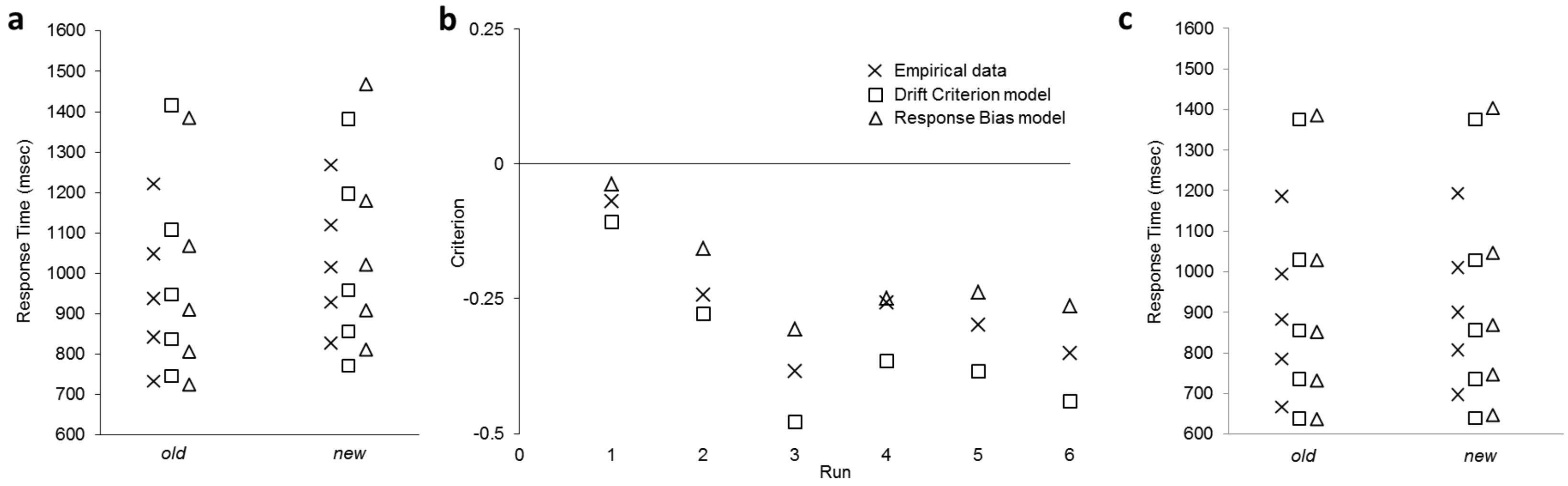

Supplementary Figure 7. **DDM posterior predictive checks**. Although model fit statistics were used to select the best-fitting model, posterior predictive checks further confirm that the best-fitting model credibly reproduces key patterns in the behavioral data. (a) Quantile plots of observed and simulated RT distributions from Experiment 1. Five RT quantiles (.1, .3, .5, .7, .9) from the empirical RT distributions are plotted for *old* and *new* responses (X's). Simulated RTs from the drift criterion DDM model (squares) and response bias DDM model (triangles) are shown for comparison. For all quantiles for both models, the respective empirical quantile value fell within the 95% credible interval of the simulated data. (b) Criterion as a function of Run, calculated for the empirical and simulated data from Experiment 1. Both models reproduce the liberal shift in criterion that occurs over the course of the experiment, and each captures the general pattern of a liberal shift that is observed in the empirical data. For each model, 500 simulated data sets were generated from the posterior distributions of model parameters, with each simulation containing 480 simulated trials for each participant. (c) Quantile plots of observed and simulated RT distributions from Experiment 2. For all quantiles for both models, the respective empirical quantile value fell within the 95% credible interval of the simulated data.

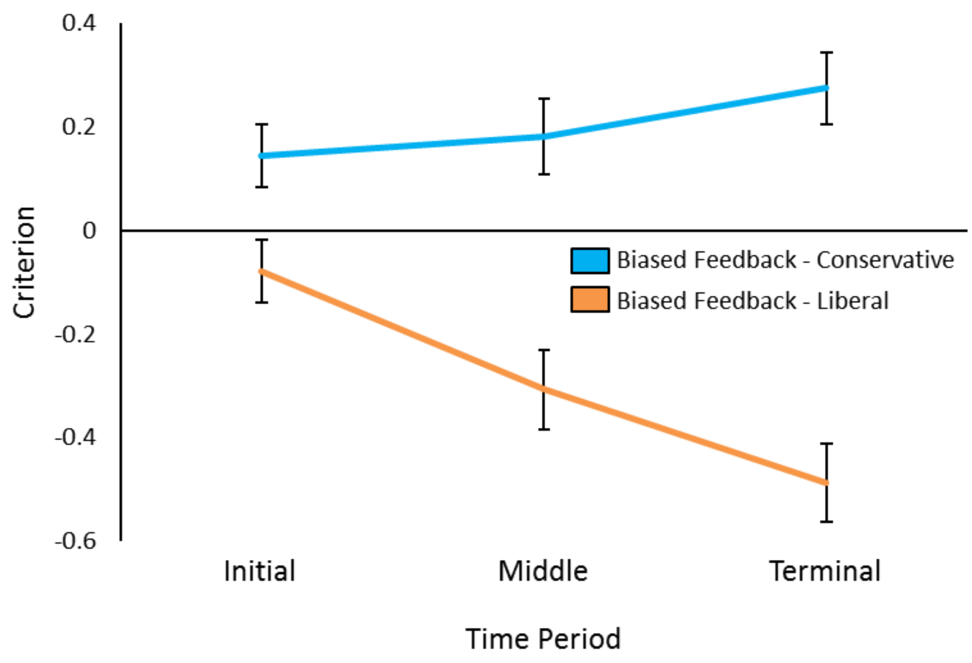

Supplementary Figure 8. Recognition criterion in the free recall transfer experiment. Participants in the Biased Feedback – Conservative and Biased Feedback – Liberal groups adopted a conservative and liberal criterion, respectively, over the course of the experiment. More liberal criterion values are plotted with negative values on the vertical axis. Error bars represent s.e.m.

Supplementary Table 1. fMRI Activations for Prediction Errors from Memory Strength and Expected Response Outcome Alternatives

Region	~Brodmann's Area	MNI Coordinates			Number of voxels	Peak Statistics	
		x	y	z		*T*	*Z*
**MS-PE Shared and Unique Variance**							
Critical cluster extent: 399							
Left hippocampus		-33	-19	-20	8542	7.95	5.15
Left precentral gyrus	6	-6	-16	49		7.32	4.92
Right caudate		6	17	1		7.18	4.87
Left amygdala		-24	2	-17		7.03	4.82
Right parietal operculum cortex	41	45	-19	19		6.89	4.76
Right supramarginal gyrus	40	57	-28	31		6.74	4.70
Left middle frontal gyrus	9	-30	26	43		6.73	4.70
Left postcentral gyrus	1	-57	-19	28		6.67	4.68
Right superior parietal lobe	5	21	-49	61		6.61	4.65
Occipital pole	18	-12	-85	16	399	6.14	4.45
**MS-PE Unique Variance**							
Critical cluster extent: 157							
Left caudate		-3	8	1	157	6.34	4.54
Right caudate		9	20	4		6.16	4.46
Left thalamus		-3	-7	10		5.76	4.28
Left postcentral gyrus	40	-45	-28	52	252	5.35	4.09
Left precentral gyrus	4	-33	-22	67		4.79	3.80

ERO-PE Shared and Unique Variance
Critical cluster extent: 126

Right superior temporal gyrus	22	57	-10	-8	7097	8.03	5.17
Left superior temporal gyrus	22	-51	-16	-2		7.74	5.07
Left precentral gyrus	6	-12	-19	70		7.59	5.02
Right precentral gyrus	6	9	-25	64		7.29	4.91
Right putamen		30	-19	4		6.98	4.80
Right cingulate gyrus	24	12	-16	43		6.71	4.69
Left medial frontal cortex	11	-9	44	-14	172	5.75	4.28
Right frontal pole	10	9	62	4		5.35	4.09
Left middle temporal gyrus	21	42	-52	10	126	5.75	4.28
Right occipital pole	18	21	-79	1	165	5.14	3.98

ERO-PE Unique Variance
Critical cluster extent: 77

Right supramarginal gyrus	40	51	-40	49	199	7.12	4.85
Right angular gyrus	40	57	-55	43		4.75	3.78
Right inferior frontal gyrus, pars opercularis	44	48	11	13	77	5.83	4.31
Left angular gyrus	40	-51	-52	49	103	5.53	4.17
Left supramarginal gyrus	40	-45	-49	37		4.35	3.55

All reported clusters were significant at a False Discovery Rate (FDR)-corrected threshold of $p < .05$ at the cluster level. Whole-brain maps were initially thresholded at $p < .001$, uncorrected, and cluster corrected to $p < .05$ using SPM’s FDR algorithm. The critical cluster extent for each contrast is listed above.

Rows that denote the peak voxel within a cluster include a value for "Number of voxels". Local maxima within the cluster reported by SPM are listed on subsequent rows.

Supplementary Table 2. fMRI Activations for Expected Value from Memory Strength and Expected Response Outcome Alternatives

Region	~Brodmann's Area	MNI Coordinates			Number of voxels	Peak Statistics	
		x	y	z		*T*	*Z*
**MS-EV Shared and Unique Variance**							
Critical cluster extent: 523							
Left precuneus	30	-9	-52	19	5051	10.67	5.92
Left frontal pole	10	-3	56	1		8.89	5.44
Left paracingulate gyrus	10	-6	56	10		8.63	5.36
Left posterior cingulate gyrus	31	-6	-43	31		8.35	5.28
Left inferior parietal lobule	7	-45	-73	31		8.12	5.20
Left medial frontal cortex	10	-3	47	-11		7.71	5.06
Left cerebellum		-18	-64	-17	523	9.31	5.56
Left inferior temporal gyrus	21	-48	-40	-14	1083	7.68	5.05
Left middle temporal gyrus	21	-57	-22	-17		7.28	4.91
Left superior temporal gyrus	22	-48	-1	-5		7.07	4.83
Left parietal operculum cortex	40	-51	-34	22		7.03	4.82
Right amygdala		30	2	-23	943	7.22	4.89
Right postcentral gyrus	40	63	-16	19		6.32	4.53
Right inferior temporal gyrus	20	57	-31	28		6.13	4.45
**MS-EV Unique Variance**							
Critical cluster extent: 42							
Left precuneus	31	-6	-64	28	566	9.79	5.70
Left posterior cingulate gyrus	31	-12	-46	34		7.99	5.16
Right posterior cingulate gyrus	31	9	-40	40		6.17	4.46
Left inferior parietal lobule	7	-42	-73	46	163	7.05	4.82

Left cerebellum		-12	-67	-14	195	7.02	4.82
Left paracingulate gyrus	32	-3	47	-2	175	6.86	4.75
Right anterior cingulate gyrus	24	6	29	10	122	6.31	4.53
Left anterior cingulate gyrus	24	-12	38	13		4.38	3.57
Anterior cingulate gyrus	32	0	-1	40	48	5.56	4.19
Right parietal operculum cortex	40	57	-31	25	42	4.60	3.69
**ERO-EV Shared and Unique Variance**							
Critical cluster extent: 130							
Left frontal pole	10	-12	62	19	2122	9.91	5.73
Left medial frontal gyrus	11	-3	47	-14		9.01	5.48
Left superior frontal gyrus	8	-15	29	64		8.67	5.37
Left middle frontal gyrus	8	-39	14	58		6.69	4.68
Paracingulate gyrus	10	0	50	4		6.30	4.52
Left orbitofrontal gyrus	11	-33	38	-14		5.64	4.23
Subcallosal cortex	32	0	26	-11		5.25	4.04
Anterior cingulate gyrus	32	0	35	4		5.05	3.93
Left posterior cingulate cortex	23	-6	-46	25	4908	9.00	5.47
Left middle temporal gyrus	21	-54	-22	-17		8.18	5.22
Left precuneus	31	-3	-64	25		7.43	4.96
Left lateral occipital cortex	18	-24	-88	1		6.88	4.76
Left superior parietal lobule	7	-24	-43	55		6.35	4.54
Left precentral gyrus	6	-3	-16	52		6.19	4.47
Right postcentral gyrus	5	12	-31	55		5.96	4.37
Right superior parietal lobule	5	21	-49	55		5.91	4.35
Left putamen		-24	-1	-2		5.72	4.26
Left inferior parietal lobule	40	-48	-67	43	666	7.60	5.02
Left inferior parietal lobule	39	-45	-70	25		6.95	4.79
Right lateral occipital cortex	19	48	-70	-8	149	7.03	4.81
Right superior temporal gyrus	21	60	-10	-8	708	6.12	4.45

Right parietal operculum cortex	13	54	-28	19		6.03	4.40
Right precentral gyrus	6	63	5	13		5.34	4.08
Right optic pole	17	18	-94	10	130	5.57	4.20
**ERO-EV Unique Variance**							
Critical cluster extent: 87							
Left frontal pole	10	-15	59	19	1277	10.18	5.80
Left medial frontal cortex	11	-3	47	-14		9.12	5.51
Left superior frontal gyrus	8	-12	29	52		6.49	4.60
Left orbital frontal cortex	11	-36	26	-20		5.73	4.27
Left orbital frontal cortex	47	-24	35	-14		5.11	3.97
Left middle temporal gyrus	21	-57	-22	-14	2590	7.79	5.09
Left posterior cingulate gyrus	23	-9	-43	34		7.40	4.95
Left superior parietal lobule	5	-30	-43	58		6.02	4.40
Left precuneus	23	-3	-64	25		5.75	4.28
Right precentral gyrus	6	6	-19	64		5.70	4.25
Right superior parietal lobule	5	27	-46	61		5.66	4.24
Left occipital pole	18	-24	-94	-5	231	7.48	4.98
Left inferior parietal lobule	39	-45	-67	40	664	7.23	4.89
Left lateral occipital cortex	39	-48	-73	19		7.23	4.89
Right lateral occipital cortex	18	48	-70	-8	306	6.54	4.62
Right parietal operculum cortex	13	54	-28	19	422	5.40	4.11
Left putamen		-24	-4	-5	87	5.16	3.99
Left cerebellum		-36	-70	-23	91	4.35	3.55

All reported clusters were significant at a False Discovery Rate (FDR)-corrected threshold of $p < .05$ at the cluster level. Whole-brain maps were initially thresholded at $p < .001$, uncorrected, and cluster corrected to $p < .05$ using SPM's FDR algorithm. The critical cluster extent for each contrast is listed above.

Supplementary Table 3. Mean Confidence Ratings for Correct and Incorrect Responses for Each Group.

	Response Type	
**Group**	**Correct**	**Incorrect**
**Veridical**	0.50 (0.06)	0.39 (0.05)
**New - High Confidence**	0.62 (0.05)	0.45 (0.04)
**New - Low Confidence**	0.54 (0.03)	0.38 (0.03)
**Old - High Confidence**	0.64 (0.04)	0.49 (0.04)
**Old - Low Confidence**	0.46 (0.03)	0.35 (0.03)

Values in parentheses represent s.e.m.

Supplementary Table 4. Comparison Between Drift Diffusion Models

Model	DIC	deviance	pD
**Experiment 1: fMRI study**			
Drift Criterion model	6457.98	6313.06	144.91
Response Bias model	6570.35	6447.43	122.92
**Experiment 2: Behavioral study**			
Drift Criterion model	22153.96	21722.42	431.53
Response Bias model	23047.51	22649.31	398.21

Lower DIC values indicate better fitting model.
DIC: deviance information criterion.

Section 3 – Supplementary Note 1

Experiment 2: Control analyses of confidence. To fully characterize the pattern of confidence responses across the various manipulations, we analyzed confidence ratings as a function of response type, time, and feedback groups. We restricted our analysis to the four groups which received false positive feedback. Confidence ratings were submitted to a five-way ANOVA with factors of recognition response (*old* / *new*), accuracy (Correct / Incorrect), time (Initial / Middle / Terminal), targeted response group (New / Old), and targeted confidence group (low / high).

A test of the Expected Response Outcome alternative requires that individuals have above-chance metacognitive accuracy; that is, correct responses are made with higher confidence than incorrect responses. This was confirmed by a main effect of accuracy ($F(1,60) = 309.343$, $p < .001$). Supplementary Table 3 shows the means (on a 0 to 1 scale) and standard error for confidence ratings of each group as a function of correct and incorrect responses. Likewise, *old* responses were made more confidently than *new* responses (main effect of Response, $F(1,60) = 35.958$, $p < .001$). Both of these effects are consistent with previous research.

There was also an accuracy by response interaction ($F(1,60) = 97.877$, $p < .001$). The difference between correct and incorrect responses was larger for *old* responses than for *new* responses. This effect was driven by the fact that correct *old* responses were made with higher confidence than correct *new* responses. This may reflect the qualitative difference between *old* responses (in which there is the presence of positive evidence for a previous encounter) and *new* responses (in which these is an absence of positive evidence for a previous encounter). It may also reflect contributions of recollection-based processes to *old* responses.

Participants in the Low Confidence groups made more low confidence responses than participants in the High Confidence groups (main effect of targeted confidence, $F(1,60) = 12.163$, $p < .001$), regardless of response type. Note that this main effect cannot explain the criterion results, because the Old – Low Confidence and New – High Confidence groups showed the largest magnitude of terminal criterion. Interestingly, this effect was not specific to the type of response that received false positive feedback (targeted confidence by feedback group interaction: $F(1,60) = 1.657$, $p = .204$). That is, this was a response–general effect: the increase in low confidence responses for the Low Confidence groups was seen across both *old* and *new* responses, regardless of which type of responses was targeted for false feedback.

Finally, there was a main effect of time such that confidence ratings became lower over the course of the experiment ($F(1.7,99.6) = 22.049$, $p < .001$); this may reflect participants' tendency to reduce their confidence ratings as they garnered more experience with the recognition task and received feedback on their performance. No other main effects of interactions reach significance after correction for multiple comparisons.

Experiment 2: Analysis of criterion as a function of time. The analysis of criterion in the main manuscript focused on the terminal criterion: the terminal time point reflects the criterion after receiving false feedback for the entire course of the experiment, and thus should best reflect the impact of feedback and PEs on criterion. Here we consider the evolution of criterion over the course of the experiment. Criterion estimates from all conditions are shown as a function of time in Supplementary Fig. 4. To assess the role of PEs on criteria, decision criterion estimates from the four false feedback conditions were submitted to a three-way ANOVA with factors of targeted response (New / Old), targeted confidence (Low / High) and time (Initial / Middle / Terminal). Across the entire experiment, we observed a main effect of false feedback: the two

New groups adopted a more conservative criterion and the two Old groups adopted a more liberal criterion ($F(1,60) = 30.551$, $p < .001$, $\eta_p^2 = .337$). We also found evidence of incremental learning: the difference between the New groups and Old groups increased over the course of the experiment (group by time interaction, $F(1.8,120) = 14.415$, $p < .001$, $\eta_p^2 = .194$).

To specifically assess the role of PEs, we next analyzed the effect of the targeted confidence manipulation. Note that any difference between the Low Confidence and High Confidence subgroups supports a role for PE-based reinforcement in regulating recognition decisions; further, the specific pattern of results allows us to discriminate between the Expected Response Outcome and Memory Strength alternatives (Fig. 5). As shown in Supplementary Fig. 4, our results support the Memory Strength alternative: the largest magnitude criteria were observed in the New – High Confidence and Old – Low Confidence groups. This alternative is supported statistically by a main effect of targeted confidence: the effect of PEs under the Memory Strength alternative manifests as a main effect because the High Confidence groups show a more positive criterion than the Low Confidence groups. Across the whole experiment, we found a main effect of targeted confidence ($F(1,60) = 4.042$, $p < .05$, $\eta_p^2 = .063$). Consistent with the Memory Strength alternative, there was no significant group by confidence interaction ($F(1,60) = 0.200$, $p = .656$, $\eta_p^2 = .003$).

We note that we did not observe a statistically reliable interaction between targeted confidence and time ($F(2,120) = 2.406$, $p = .095$). Because false feedback is provided immediately from the onset of the test phase, even the criterion estimate during the *initial* time period reflects the influence of feedback on an individual's criterion. Indeed, in many decision-making and reinforcement learning tasks, learning rate is often highest during the initial portion of a learning experience. Because the experiment was double blind and employed random

assignment, we have no reason to assume that there were group differences in default criterion before participants began the experiment. Thus, we interpret the main effect of targeted confidence as evidence that the false feedback manipulation caused shifts in criterion from individuals' pre-experimental default criterion. However, we cannot rule out the possibility that our effect is due to a failure of random assignment (a Type I error with probability equal to our alpha level of 0.05). We did observe an interaction between targeted confidence and time for the two New groups ($F(2, 60) = 5.020$, $p < .01$) but not for the two Old groups ($F < 1$). This null result may be due to rapid learning in the Old – Low Confidence condition that resulted in a large criterion shift in the initial phase (Supplementary Fig. 4).

Experiment 2: Individual differences analysis of Net PE metric. The between-groups analyses demonstrate the causal effect of the experimental manipulation (targeted false positive feedback) on criterion. However, individuals do not only experience PEs following false positive feedback; instead, our framework suggests that each feedback instance provides a PE signal. To further assess the role of Memory Strength PEs (MS-PE) in regulating criterion, we computed the Net MS-PE that each participant encountered across all trials of the recognition test, and correlated this Net MS-PE with each participant's criterion. Thus, this analysis allows us to leverage the total PE signal that an individual participant encounters across all trials, including both veridical and false feedback trials.

Using the logic of Fig. 1, we calculated a Memory Strength PE for each trial (see Methods for quantitative details). To match the sign of criterion estimates (more liberal criterion is more negative), the sign of PEs was adjusted such that all PEs favoring *old* responses (positive outcomes after an *old* response; negative outcomes after a *new* response) were given a negative sign, while all PEs favoring *new* responses (positive outcomes after a *new* response; negative

outcomes after an *old* response) were given a positive sign. For each participant, these PEs were then averaged across all trials (including trials that received false feedback and trials that received veridical feedback) to assign each participant a Net MS-PE. If criterion is regulated through PE-based learning, more negative Net MS-PE scores should be associated with more liberal criteria and more positive Net MS-PE scores should be associated with more conservative criterion.

Supplementary Fig. 5a shows the correlation between Net MS-PE scores and terminal criterion for the four false feedback groups. However, this relationship also includes the group effect of targeted response: that is, it may simply be the case that because the two New groups received more positive feedback on *new* responses and adopted a conservative criterion (and vice versa for the Old groups), this group effect is driving the correlation (*cf*. Simpson's Paradox). In order to demonstrate the effect of Net MS-PEs over and above the effect of targeted response group (New / Old), we performed a multiple regression analysis that included a categorical predictor for Group (participants in the two New groups were coded as 1 and participants in the two Old groups were coded as 0) and a predictor for Net MS-PE. In this model, the regression weight for the Net MS-PE predictor represents partial correlation coefficients controlling for the effect of Group. This model provided a reliable fit to the data (R = 0.679; $p < .001$) and the Net MS-PE was a significant predictor of terminal criterion ($p = .034$). Supplementary Fig. 5b depicts a partial regression plot which shows the residuals from regressing Terminal Criterion against Group on the vertical axis against the residuals from regression Net MS-PE against Group on the horizontal axis. The correlation between these two sets of residuals shows the linear relationship (partial correlation) between Terminal Criterion and Net MS-PE in the multiple regression controlling for the effect of Group. This regression model indicates that Net

MS-PE predicts criterion even after controlling for the effect of group, consistent with a role of PE-based learning that occurs for both veridical and false feedback group.

Finally, we used this individual differences approach to assess whether the Net MS-PE signal provides a better fit to the data than a Net Expected Response Outcome PE (ERO-PE) signal. For each trial, we calculated the ERO-PE (see Fig. 1; Methods) and then transformed the sign of this PE signal to match the effect on criterion (as described above). For each participant, we then took the mean of the ERO-PE across all trials to compute the Net ERO-PE. We performed a multiple regression analysis that included predictors for group, Net MS-PE, and Net ERO-PE. This model tests for three partial correlation coefficients: (1) the effect of Group, after controlling for Net MS-PE and Net ERO-PE; (2) the effect of Net MS-PE, after controlling for Group and Net ERO-PE; and (3) the effect of ERO-PE, after controlling for Group and Net MS-PE. In this model, however, we find that none of the individual predictors reach significance (group: $p = .228$; MS-PE: $p = .083$; ERO-PE: $p = .258$). That is, none of the individual predictors provides a reliable increase in predictive power after controlling for the other two predictors in the model. However, the reliable multiple correlation coefficient for the model ($R = 0.688$; $p < .001$) indicates that the model, as a whole, is a strong predictor of terminal criterion. In summary, the individual differences analyses provide evidence that the Net MS-PE metric predicts terminal criterion over and above the effect of group, but they do not provide additional evidence that favors the Memory Strength alternative over the Expected Response Outcome alternative.

Section 4 - Supplementary Methods

Experiment 2: Adaptive feedback details and manipulation checks. The false feedback algorithm was motivated by a pilot experiment in which we preferentially targeted false feedback towards errors made with confidence ratings either below (Low) or above (High) the midpoint of the confidence range (the point half-way up the rectangle used to indicate confidence). For example, a participant in the Old – Low Confidence group would receive false positive feedback on 100% of *old* response errors made with a confidence below the midpoint, false positive feedback on 40% of *old* response errors made with a confidence above the midpoint, and veridical feedback on all other trials. In contrast, a participant in the Old –High Confidence group would receive false positive feedback on 40% of *old* response errors made with a confidence below the midpoint, false positive feedback on 100% of *old* response errors made with a confidence above the midpoint, and veridical feedback on all other trials. Participants were instructed to try to use the entire range of the confidence scale. If *old* errors were made with an average confidence of 0.5 and were normally (or equally) distributed across the confidence range, this would result in false positive feedback on 70% of *old* errors for both hypothetical participants, but a higher average level of confidence for false feedback trials in the High Confidence group.

However, a manipulation check found that this approach did not lead to reliable differences in the average confidence of trials targeted for false feedback; that is, the average confidence targeted for false feedback in the High Confidence groups was not reliably different than the Low Confidence groups. Inspection of the data suggested that this manipulation failure was due to individual differences in error rates and in the way that participants used the confidence scale. For example: some participants, on average, were more likely to use the

portion of the confidence scale above the midpoint. If this participant were assigned to a Low Confidence group, she would not be provided many instances of false feedback.

Thus, the method for targeting false positive feedback used in the present experiment employed an adaptive algorithm that took into account individual differences in confidence ratings and error base rates. This information was used to provide approximately equal instances false feedback to each participant, provide the false feedback equally across the course of the entire recognition test phase, and maximize the difference in the targeted confidence between subgroups. The experimental script was designed to provide 30 false feedback trials to each participant. For example, consider a participant receiving false feedback targeted to *old* errors whom the script estimated would make 36 instances of *old* errors. Approximately 83% of her *old* errors would receive false positive feedback, in order to provide 30 total instances of false feedback. For the first four error trials, the script assumed an error rate of 36 errors (approximately equal to the error rate seen in pilot data). For the fifth and subsequent errors, the script used the error rate determined on-line specific to the participant.

The experimental script also tracked the distribution of confidence used for each response type (*old* or *new*), on a participant-by-participant basis. This distribution was used to determine if a given trial would receive false feedback. If the script was aiming to provide false feedback on *X* percent of the remaining error responses, the script would determine whether the participant's confidence on a given trial fell in either the lowest or highest *X* percent of the participant's confidence distribution, depending on the condition. This distribution and cut-off point *X* was updated on a trial-by-trial basis for each participant.

Continuing the previous example, the script might determine that 83% of the remaining *old* response errors should receive false feedback. For a participant in the Old – High Confidence

group, an *old* response error given confidence rating above the 17th percentile mark in her specific confidence distribution would receive false positive feedback. An *old* error given a confidence rating below the 17th percentile mark in her confidence distribution would receive veridical negative feedback. This would ensure that false feedback was provided with the appropriate frequency (as estimated by the script) and preferentially targeted to high but not low confidence *old* errors for this participant. Conversely, for a participant in the Old – Low Confidence group, an *old* error given a confidence rating below the 83rd percentile received false positive feedback; an *old* error given a confidence rating above the 83rd percentile mark received veridical negative feedback.

The feedback manipulation was successful at targeting false feedback to high or low confidence responses. The count of false feedback trials was submitted to a targeted response (Old / New) by targeted confidence (Low / High) ANOVA. The average targeted confidence for Low Confidence groups was significantly lower than for the High Confidence groups (Means = 0.26, 0.62; $F(1,60) = 95.860$, $p < .001$). The experimental script was designed to provide 30 false feedback trials to each participant; the mean number of false feedback trials was 33.1. This deviation was likely to do participants proclivity to make increased errors (due to the false feedback manipulation) over the course of the experiment, such that the experimental script initially underestimated participants' total error rates and subsequently provided additional false feedback trials.

There was a significant difference in the number of false feedback trials between the Low and High Confidence groups ($F(1,60) = 6.927$, $p < .05$) such that participants in the Low Confidence groups were provided more false feedback than the High Confidence groups (Means: Old – Low Confidence: 37.4; Old – High Confidence: 29.4; New – Low Confidence: 34.1; New

– High Confidence: 31.6). This is consistent with the idea that errors are generally made with lower confidence. Note that this difference alone cannot explain the pattern we report in the recognition criterion, as the larger magnitude criteria were shown by the Old – Low Confidence group and the New – High Confidence group.
